# The SET-Domain Protein SUVR5 Mediates H3K9me2 Deposition and Silencing at Stimulus Response Genes in a DNA Methylation–Independent Manner

**DOI:** 10.1371/journal.pgen.1002995

**Published:** 2012-10-11

**Authors:** Elena Caro, Hume Stroud, Maxim V. C. Greenberg, Yana V. Bernatavichute, Suhua Feng, Martin Groth, Ajay A. Vashisht, James Wohlschlegel, Steve E. Jacobsen

**Affiliations:** 1 Department of Molecular, Cell, and Developmental Biology, University of California Los Angeles, Los Angeles, California, United States of America; 2 Department of Biological Chemistry, David Geffen School of Medicine, University of California Los Angeles, Los Angeles, California, United States of America; 3 Howard Hughes Medical Institute, University of California Los Angeles, Los Angeles, California, United States of America; National Cancer Institute, United States of America

## Abstract

In eukaryotic cells, environmental and developmental signals alter chromatin structure and modulate gene expression. Heterochromatin constitutes the transcriptionally inactive state of the genome and in plants and mammals is generally characterized by DNA methylation and histone modifications such as histone H3 lysine 9 (H3K9) methylation. In *Arabidopsis thaliana,* DNA methylation and H3K9 methylation are usually colocated and set up a mutually self-reinforcing and stable state. Here, in contrast, we found that SUVR5, a plant Su(var)3–9 homolog with a SET histone methyltransferase domain, mediates H3K9me2 deposition and regulates gene expression in a DNA methylation–independent manner. SUVR5 binds DNA through its zinc fingers and represses the expression of a subset of stimulus response genes. This represents a novel mechanism for plants to regulate their chromatin and transcriptional state, which may allow for the adaptability and modulation necessary to rapidly respond to extracellular cues.

## Author Summary

The ability of eukaryotic cells to respond to external stimuli depends on the coordinated activation and repression of specific subsets of genes, often relying on chromatin structure modification. Here, we have characterized a locus-specific mechanism to repress gene expression by the action of an *Arabidopsis thaliana* SET domain protein, SUVR5, the first example of sequence-dependent heterochromatin initiator in the plant kingdom. Our results suggest that SUVR5 establishes the heterochromatic state by H3K9me2 deposition in a DNA methylation–independent manner that is not perpetuated and thus allows for changes in response to the environment or developmental cues.

## Introduction

In eukaryotes, chromatin structure regulates the access of the transcriptional machinery to genetic elements, playing an important role in the regulation of gene expression. The transition between transcriptionally active (loosely packed) chromatin and repressed (tightly packed) chromatin states is controlled by covalent modifications of the histone tails, DNA cytosine methylation, and the differential use of histone variants [1]. In mammals and plants, transcriptionally inactive chromatin—or heterochromatin—is typically associated with DNA methylation and histone H3 lysine 9 methylation (H3K9me). These epigenetic silencing marks are generally thought to be coordinately regulated by cooperation between DNA methyltransferases and histone methyltransferases, contributing to their stability and self perpetuating nature. However, in order to readily adapt to environmental stimuli or developmental cues, some of these marks also need to be reversible, although how this is achieved is currently unclear.

Most histone methyltransferases (HMTases) contain a catalytic SET domain (named after three *Drosophila* proteins: Suppressor of position effect variegation 3–9, SU(VAR)3–9; Enhancer of zeste, and Trithorax) [2]. The enzymatic activity of the SET domain was first discovered in a mammalian homolog of SU(VAR)3–9, SUV39H1, which was shown to methylate histone H3 at lysine 9 [3]. In plants, there is a relatively large family of SET domain-containing proteins that are closely related to Drosophila SU(VAR)3–9 and its human and *S. Pombe* homologs (SUV39H and CLR4, respectively) [4]. In *Arabidopsis thaliana*, of the 14 SET domain-containing proteins most related to SU(VAR)3–9, nine are classified as SU(VAR)3–9 HOMOLOGS (SUVH1–SUVH9), and five as SU(VAR)3–9-RELATED proteins (SUVR1–SUVR5). Arabidopsis SUVH proteins link the epigenetic silencing marks H3K9me2 and DNA methylation through the activity of their SRA domains (for SET and RING finger Associated), which bind different contexts and states of methylated DNA. Contrary to SUVHs, most of the SUVR proteins are of completely unknown function. In addition, because they lack the SRA domain, how they are recruited to chromatin is unknown.

In Arabidopsis, DNA methylation occurs in three different sequence contexts: CG, CHG and CHH (where H is any base other than G). In all cases, *de novo* DNA methylation is established by DOMAINS REARRANGED METHYLTRANSFERASE 2 (DRM2), a homolog of the mammalian DNA METHYLTRANSFERASE 3 (DNMT3) family [5]. Subsequent to establishment, DNA methylation is maintained through the cell cycle by at least three different pathways depending on the sequence context [6]. The maintenance of CHH methylation is mostly carried out by DRM2 through persistent *de novo* methylation [6], [7]. The maintenance of CG methylation depends on METHYLTRANSFERASE 1 (MET1), the Arabidopsis homolog of mammalian DNA METHYLTRANSFERASE 1 (DNMT1), in collaboration with the VARIANT IN METHYLATION/ORTHRUS (VIM/ORTH) family [8], [9], [10], the Arabidopsis homologs of the mammalian UHRF1. These proteins contain SRA domains that bind to hemimethylated CG sites [11], [12], [13].

The maintenance of CHG methylation relies on CHROMOMETHYLASE 3 (CMT3), a plant specific DNA methyltransferase that acts together with some of the above mentioned SUVH proteins, KRYPTONITE (KYP)/SUVH4, SUVH5, and SUVH6 [14], [15], [16], [17], which can bind directly to methylated-DNA [11], [18]. The structure of the SUVH5 SRA domain bound to methylated DNA has been solved revealing that two SRA domains bind independently to each strand of the DNA duplex at either a fully or hemimethylated site [19]. These data support a model where regions rich in DNA methylation serve as binding platforms for KYP, SUVH5 and/or SUVH6, leading to H3K9 methylation. Histone methylation would then provide a binding site for CMT3 via its chromodomain, leading to CHG methylation, and thus creating a purely epigenetic self-reinforcing feedback loop for the maintenance of DNA and histone methylation, which explains the stability of epigenetic silent states and their self perpetuating nature [11].

The link between H3K9 methylation and DNA methylation is further supported by the strong genome-wide correlation between heterochromatic H3K9me2 and DNA methylation [20]. In addition, *kyp* mutants show decreased levels of both H3K9me2 and cytosine methylation [14], [21], [22], which are even further reduced in higher order *suvh* mutants [16], [17]. Moreover, loss of DNA methylation in *met1* mutants correlates with a global loss of H3K9me2 [22].

In this report we show that Arabidopsis SU(VAR)3–9 RELATED 5 (SUVR5), which lacks the SRA domain present in its SUVH counterparts, is able to recognize specific DNA sequences through a DNA binding domain that contains three zinc fingers, and induce silencing through DNA-methylation independent H3K9me2 deposition, possibly acting as part of a histone modifier multimeric complex. We propose that SUVR5 mediates a mechanism for heterochromatin formation that is distinct from the self-perpetuating loop existing between H3K9me2 and DNA methylation, and that this lack of perpetuation allows for the increased plasticity needed in response to environmental or developmental cues during an organism's life.

## Results

### SUVR5 is important for plant development and contains a zinc finger domain that binds to DNA

**Figure 1:**
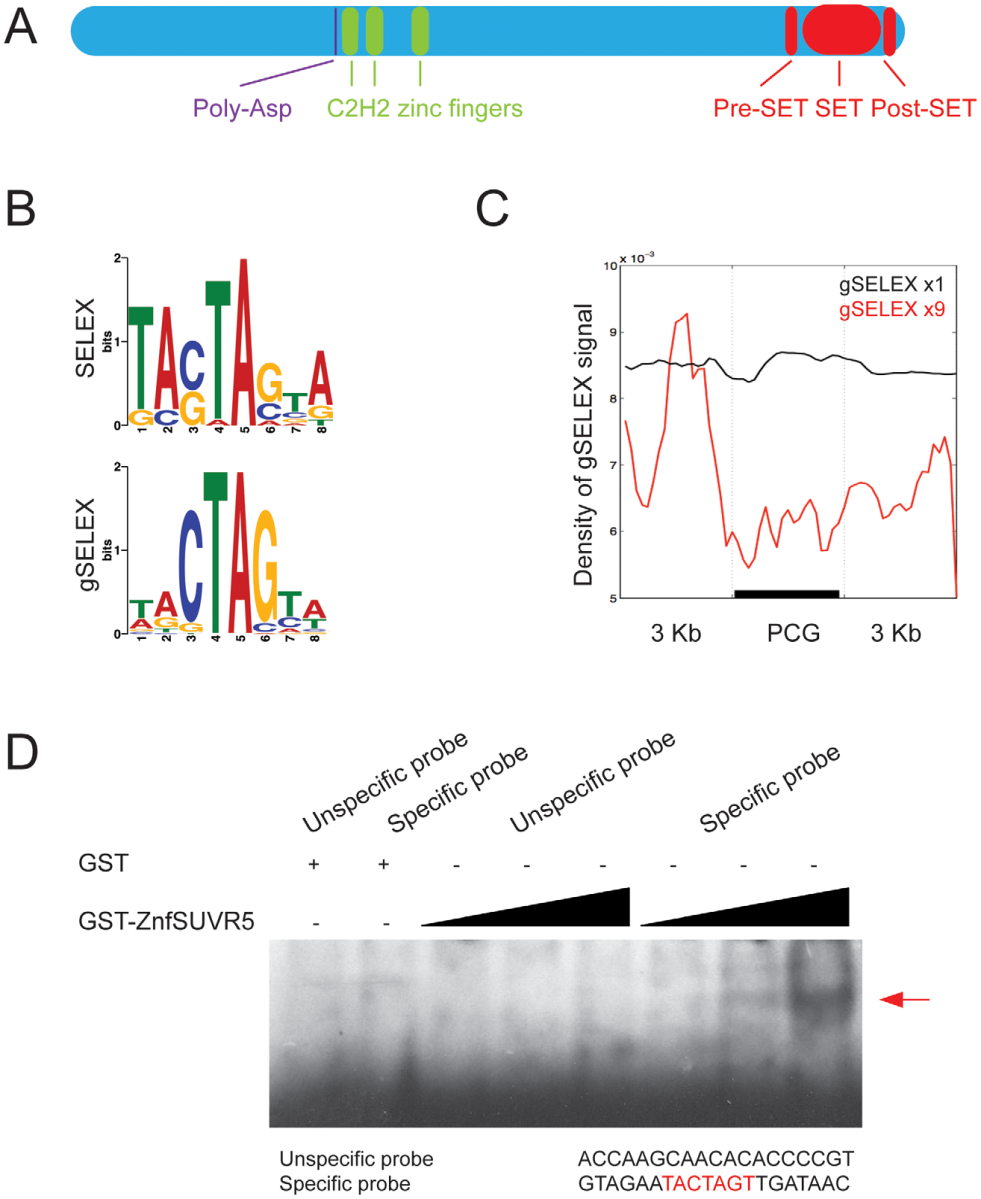
SUVR5 zinc finger domain binds specific sequences of DNA that map to the promoters of genes. a, Domain structure of SUVR5 (Poly-Asp: domain of unknown function rich in Asp residues); b, enriched motifs identified in the sequencing data obtained from the SELEX experiments; c, meta-gene analysis of the genomic SELEX (gSELEX) reads showing preferential binding of the SUVR5 zinc finger domain to the 3 Kb region upstream of protein coding genes (PCG). The results obtained after exponential selection of the binding sites for 9 cycles are shown (×9) in contrast with the results obtained after only one cycle of enrichment (×1), included as control; d, mobility shift assay with increasing amounts of GST-zinc finger domain (100, 250 and 500 ng) added to a binding reaction with either an unspecific oligonucleotide probe or a specific probe including the identified binding motif sequence.

To test the role of Arabidopsis SU(VAR) 3–9 RELATED genes in plant development we screened T-DNA mutants in all five *suvr* single mutants and higher order combinations for visible morphological defects. We found that the *suvr5-1* mutation produces a delay in flowering time that was not further enhanced in the quintuple *suvr1 suvr2 suvr3 suvr4 suvr5* mutants (Figure S1). These observations were consistent with results from earlier analysis of a *suvr5* mutant [23] and suggested a role for SUVR5 (but not the other SUVR family members) in flowering time. SUVR5 differs from the other SUVR family members in that it contains a set of three C2H2 zinc fingers in tandem in the central part of the protein (Figure 1a). SUVR5 homologs with a similar domain architecture (zinc fingers plus a C-terminal SET domain) are found in all plant species analyzed suggesting that it is widely conserved in the plant kingdom (Figure S2). We hypothesized that the zinc fingers have a DNA-binding function and may direct SUVR5 epigenetic activity to sequence-specific regions of the genome. To test this, we used the Systematic Evolution of Ligands by Exponential Enrichment (SELEX) technique with the recombinant SUVR5 zinc fingers domain to analyze binding to oligonucleotides that included a 15 base-pair (bp) random sequence (Figures S3 and S4). We identified an 8-nucleotide motif favored by SUVR5 binding (Figure 1b, upper panel). Next, we repeated the experiment using 100 bp fragmented Arabidopsis wild-type Col-0 genomic DNA (genomic SELEX, gSELEX) to identify naturally occuring SUVR5 binding sequences (Figure S5). We identified almost the exact same binding motif “TACTAGTA” (Figure 1b, lower panel)—a palindromic octamer that is consistent with the 9-nucleotide that is the maximum expected size of a sequence recognized by three zinc fingers in tandem, since each zinc finger repeat has a predicted alpha-helical core that binds to 3 nucleotides in the major groove of DNA [24]. The binding and its specificity were confirmed by electromobility shift assays (EMSAs) (Figure 1d, Figure S6).

The high throughput sequencing results from the genomic SELEX experiment allowed us to map the identified SUVR5 binding regions to the Arabidopsis genome. Metaplot analysis showed that these regions mapped preferentially to the area immediately upstream of transcriptional start sites of protein coding genes (Figure 1c).

### SUVR5 affects H3K9me2

Given the SUVR5 SET domain homology to *Drosophila* SU(VAR)3–9 we hypothesized that SUVR5 is an active methyltransferase. Consistent with this, SUVR5 bound to the methyl-group donor SAM (Figure S7) and its SET domain contains all of the crucial residues required for histone methyltransferase activity in the HΦΦNHSC motif. However, we were unable to demonstrate *in vitro* histone methytransferase activity against various histone substrates. This could indicate that other binding partners are necessary for SUVR5 enzymatic activity, similar to other histone methyltransferase complexes such as those containing Enhancer of Zeste [25], or that SUVR5 biochemical activity is dependent on a particular chromatin context [26].

**Figure 2:**
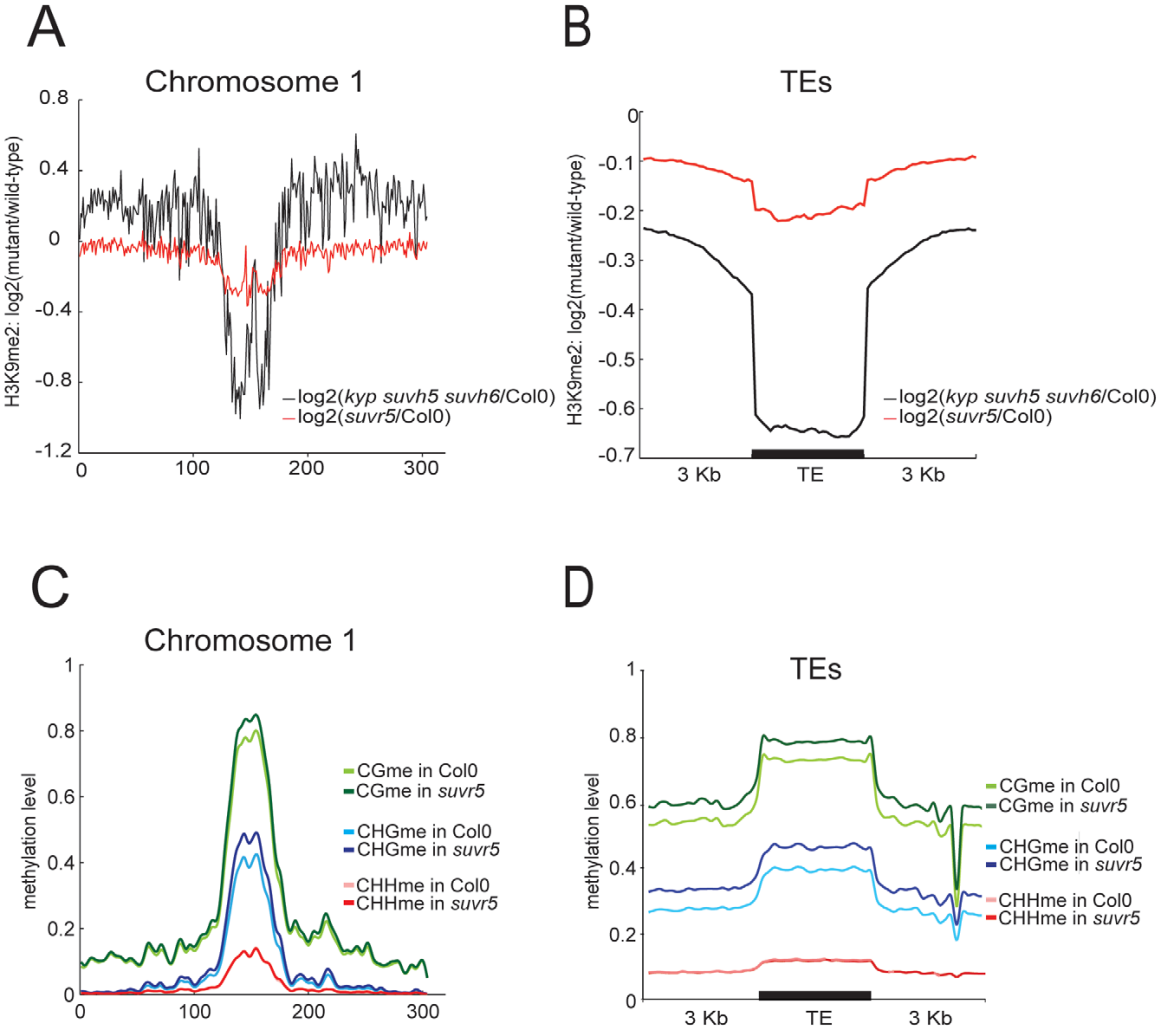
SUVR5 is redundant with KYP/SUVH5/SUVH6 in controlling H3K9me2 accumulation in heterochromatin. a, Chromosome 1 view of the log2 ratio of H3K9me2 signal in *suvr5* mutants vs. Col-0 (red), and the log2 ratio of *kyp suvh5 suvh6* triple mutants vs. Col-0 (black); b, Chromosome 1 distribution of DNA methylation in *suvr5-1* and Col-0; c, meta-analysis of H3K9me2 levels on *suvr5* and *kyp suvh5 suvh6* mutants vs. Col-0 over TEs; d, meta analysis of CG, CHG and CHH DNA methylation levels in *suvr5-1* and Col-0 over TEs. (green = CG, blue = CHG, red = CHH; light colors are Col-0, and dark colors are *suvr5-1*).

We directly tested for the role of SUVR5 on H3K9me2 levels *in vivo* by utilizing chromatin immunoprecipitation followed by microarray analysis (ChIP-chip) experiments in mature leaves of wild-type Col-0 and *suvr5-1* mutants. The *suvr5* mutants showed an overall decrease in H3K9me2 accumulation on pericentromeric heterochromatin (Figure 2a, Figure S8) and transposable elements (TEs) (Figure 2b), although these effects were relatively minor. Heterochromatic H3K9me2 is known to be mostly maintained by KYP, SUVH5 and SUVH6 [14], [16], [17], [21], [22], and ChIP-chip data with the *kyp suvh5 suvh6* triple mutants showed a much more dramatic decrease in H3K9me2 levels than with *suvr5* (Figure 2a and 2b). These data confirm that KYP, SUVH5, and SUVH6 are the major H3K9m2 enzymes in heterochromatin, but also suggest a minor role for SUVR5.

H3K9me2 is correlated with DNA methylation in Arabidopsis on a genome wide level [20]. The loss of H3K9me2 in *kyp* mutants produces a decrease in DNA methylation [14], [21], [22] that is enhanced in the *kyp suvh5* or *kyp suvh6* double mutants and in the *kyp suvh5 suvh6* triple mutant [16], [17]. Importantly, in the case of *suvr5* mutants, we did not detect a decrease in DNA methylation at pericentromeric heterochromatin (Figure 2c, Figure S9) or TEs (Figure 2d, Figure S10), suggesting that SUVR5 functions differently than the SUVH proteins.

**Figure 3:**
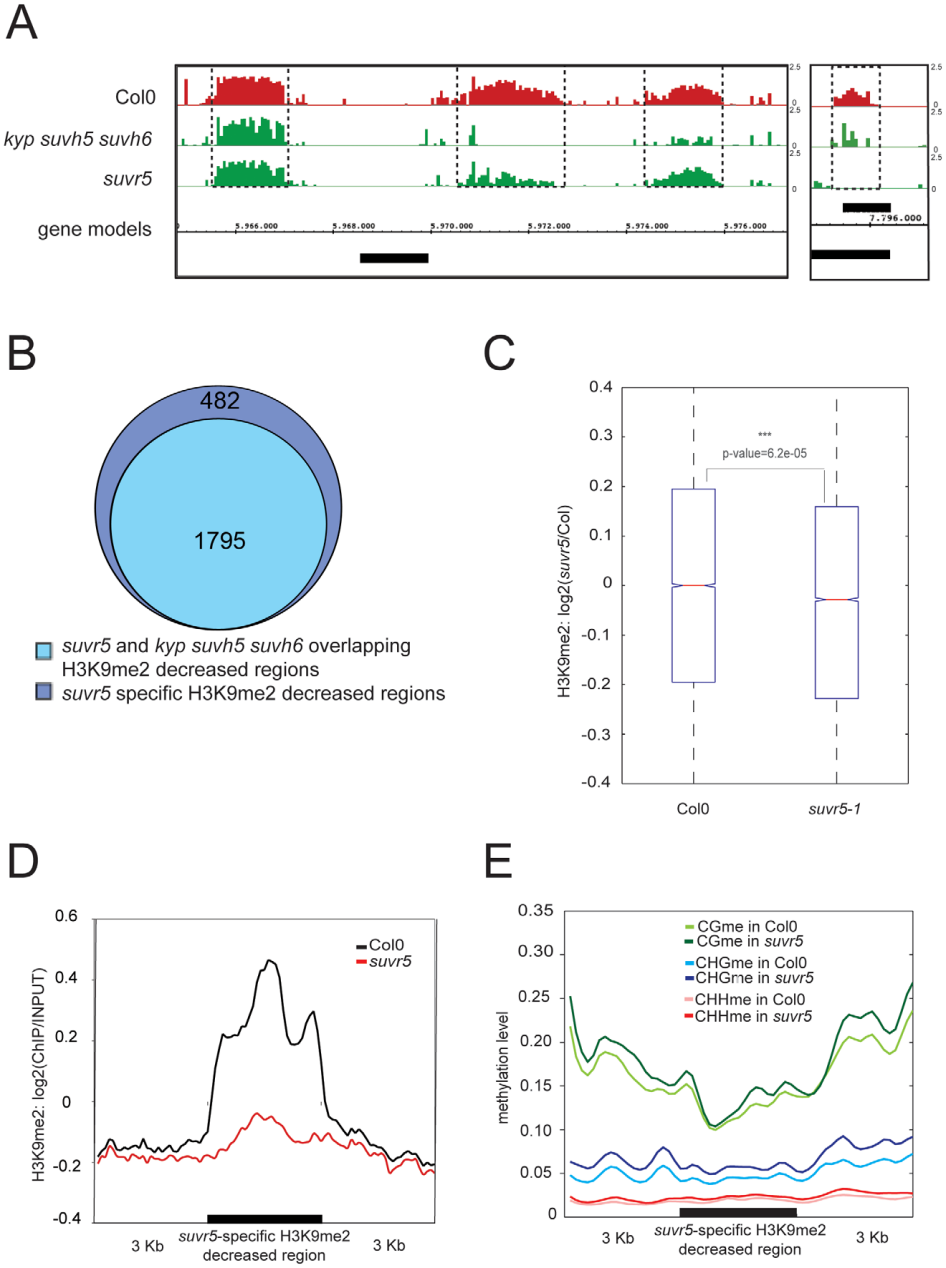
SUVR5-specific H3K9me2 deposition correlates with the zinc finger domain binding and promotes gene silencing. a, genome browser view of a region in the arms of chromosome 1. H3K9me2 data is represented as log2 ratios from 0 to 2.5. Gene models correspond to TAIR8 protein-coding genes (PCG) and are shown for the plus or minus strand of the genome; b, Venn diagram representation of the number of H3K9me2 decreased regions defined for *suvr5* mutants that are specific to them or overlap with the ones in *kyp suvh5 suvh6*; c, box plot showing the levels of H3K9me2 in the genes that have gSELEX signal in their upstream 3 Kb region; d, meta-analysis of H3K9me2 levels on *suvr5-1* and Col-0 over the *suvr5*-specific H3K9me2 decreased regions; e, meta analysis of CG, CHG and CHH DNA methylation levels in *suvr5-1* and Col-0 over the *suvr5*-specific H3K9me2 decreased regions.

We could also detect regions within the arms of the chromosomes with a decrease in H3K9me2 levels in the *suvr5* mutants. Although the majority of these regions overlapped with regions dependent on KYP/SUVH5/SUVH6, over 20% were specific to *suvr5* (Figure 3a and 3b). These *suvr5*-specific regions consisted of discrete patches of H3K9me2 that were solely dependent on SUVR5 (Figure 3d), and were characterized by very low levels of cytosine DNA methylation, and these levels of DNA methylation were not altered by the loss of *SUVR5* (Figure 3e). These results suggest that, in those specific locations, SUVR5 is controlling H3K9me2 deposition in a DNA-methylation-independent manner that is not perpetuated by the KYP/CMT3 epigenetic loop. We could also find a small number of transposons in the chromosome arms whose H3K9me2 decrease was specific for *suvr5* mutants and independent of *kyp/suvh5/suvh6,* and these tended to be smaller transposons with lower levels of DNA methylation (Figure S11). We analyzed for the presence of SUVR5 binding motifs within the sequence of these 423 TEs that show decreased levels of H3K9me2 specifically in *suvr5* mutants ±2 Kb and 8.5% of them contain the motif TACTAGTA.

To determine if there is a correlation between H3K9me2 levels and SUVR5 binding, we analyzed H3K9me2 levels in the set of genes that were shown to bind the SUVR5 zinc fingers (i.e. with signal 3 Kb upstream of their transcription start site) in the gSELEX experiment. In that specific set of genes, we found a significant decrease of H3K9me2 when comparing *suvr5* mutants to wild-type (Figure 3c). This decrease was significant for both of the ChIP-chip replicates analyzed (Figure S12). Analysis of all the genes that show a H3K9me2 decrease in *suvr5* mutants compared to wild type showed that around 27% of them have gSELEX signal in their proximal promoter (1 Kb upstream their TSS). Interestingly, when we analyze not only euchromatic regions, but all decreased H3K9me2 regions including those in pericentromeric heterochromatin, only 5.4% of them overlap with the gSELEX signal. This suggests that targeting of SUVR5 to pericentromeric heterochromatin may be mediated by another unknown mechanism, which is likely responsible for the redundancy of SUVR5 with KYP/SUVH5/SUVH6.

### Biological relevance of SUVR5 function

To measure the effects of SUVR5 on gene expression, we performed mRNA sequencing (mRNA-Seq) experiments to analyze the transcriptome of *suvr5-1* mutants. We observed a large number of genes that were signficantly upregulated, the majority of which were located in the euchromatic chromosome arms (Table S1, Figure S13). Although many of these genes are likely to be indirect targets to SUVR5, 11% of these genes were among those that showed decreased H3K9m2 levels, and 69.5% of these genes contained at least one significant SUVR5 binding motif in their promoter. Examples of genes with a decrease in H3K9me2 levels and upregulated expression in two different alleles of *suvr5* mutants can be found in Figure S14 (See Figure S15 for *suvr5-2* mutant allele characterization). Consistent with the slight decrease of H3K9me2 levels that occurred in *suvr5* at TEs, very few transposons were reactivated in the mutant (Table S2).

**Figure 4:**
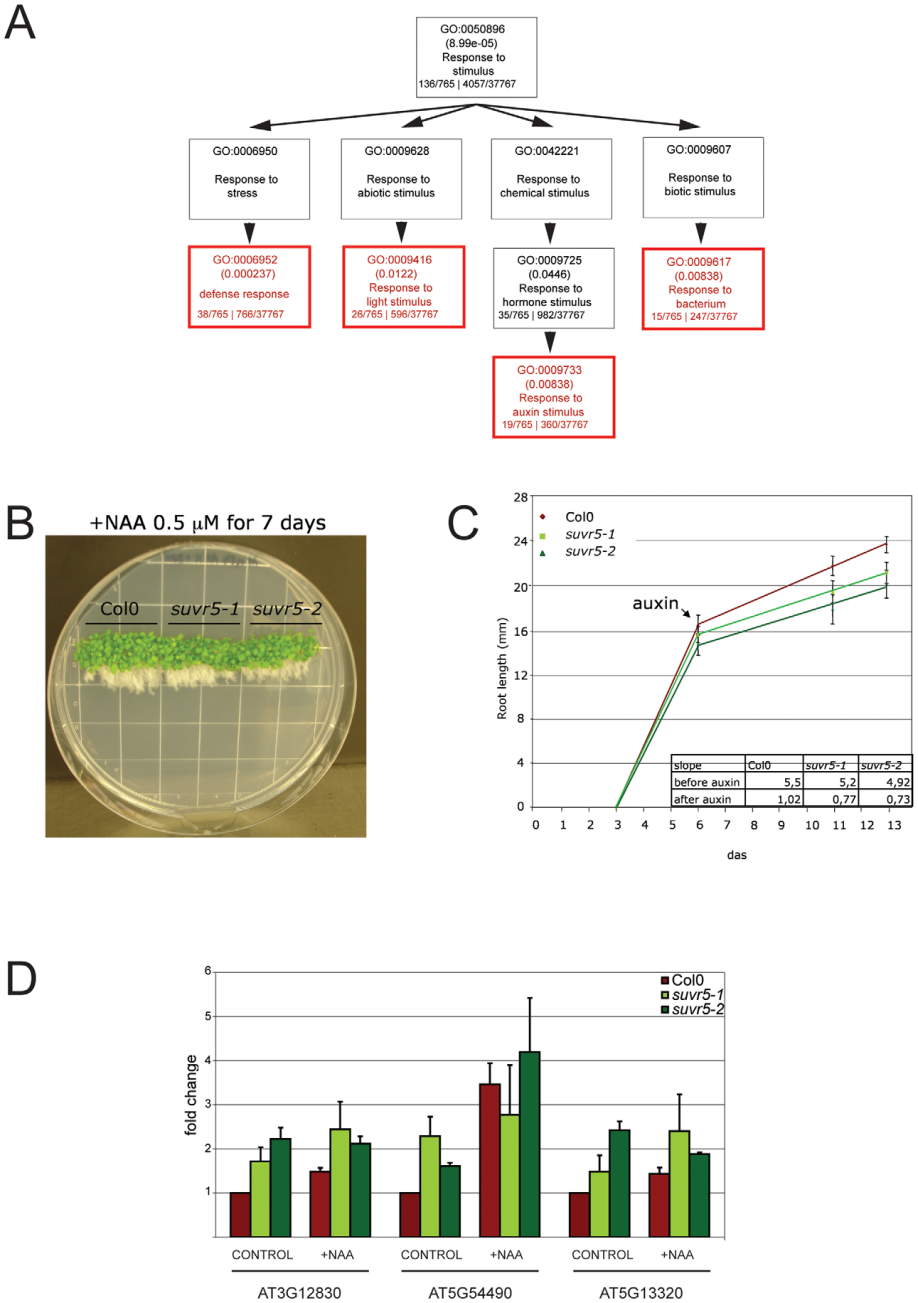
SUVR5 significantly affects the expression of genes related to the “response to stimulus” GO term. a, chart showing the GO term “response to stimulus” category and subcategories under it and the level of significance of their over-representation in the set of *suvr5* upregulated genes compared to the whole genome (p-values shown in parentheses). At the bottom of each box, the number of genes that include the particular GO term in the *suvr5* upregulated set of genes/total number of *suvr5* upregulated genes is shown on the left; the number of genes that include the particular GO term in the whole genome/total number of genes in the whole genome is shown on the right; b, picture of Col-0, *suvr5-1* and *suvr5-2* 13-day-old seedlings treated with 0.5 µM NAA for the last 7 days. Notice the differences in growth; c, time course root length measurements of Col-0, *suvr5-1* and *suvr5-2* seedlings before and after NAA treatment. The bottom right panel shows the slopes of the curves that represent a measurement of the growth rate. Around 20 seedlings of each line were measured and SE is shown for every point; d, expression levels (relative to *ACTIN*, relative to Col-0 CONTROL) of 3 selected auxin-responsive genes in seedlings grown for 12 day without auxin (NAA) application (CONTROL) or transfered to NAA media on the fifth day (+NAA 0.5 µM). The experiment was done in triplicate and SE is shown for each sample.

To identify the biological processes that SUVR5 may regulate, we applied gene ontology (GO) term analysis to the genes upregulated in the *suvr5* mutant (over 4 fold, p-value<0.01). Of the three broad GO term categories significantly over-represented in this set of genes, the most significantly enriched was “response to stimulus” (Figure S16). This category includes subcategories such as defense response, response to biotic stimuli like bacterium, and response to endogenous stimuli like the plant hormone auxin, which were strongly and significantly enriched (p_value<0.01; Figure 4a).

Auxin plays a key role in many plant developmental processes [27], [28]. For example auxin plays a central role in elaborating root architecture because of its role in endogenous developmental programs as well as its mediation of environmental stimuli responses [29]. We hypothesized that the overexpression of auxin inducible genes in *suvr5* mutants might generate a partially constitutive auxin-response in the abscence of the hormone. Auxin causes inhibition of root growth by reduction of cell division and elongation, and a constitutive response could explain the defects in root growth earlier reported for *suvr5* mutants [30], which we also observed here for both of the *suvr5* alleles tested (Figure 4b and c). To examine this, we analyzed the expression of three examples of genes annotated as “auxin-responsive” and that have significant SUVR5 binding sites in their promoters (Figure S17). These genes are annotated as a PINOID (PID)-binding protein (At5g54490), an auxin-responsive GH3 family protein (At5G13320), and a SAUR-like auxin-responsive family protein (At3g12830). We found that these genes were indeed upregulated upon auxin treatment (Figure 4d) and that in the *suvr5* mutants, these genes also showed increased expression, even in the absence of the hormone (Figure 4d). These data are consistent with a model whereby a stimulus such as auxin treatment overcomes the repression established by SUVR5, activating the genes and thus guaranteeing an appropriate response to environmental and developmental cues.

### Interaction of SUVR5 with the LDL1 histone demethylase

**Figure 5:**
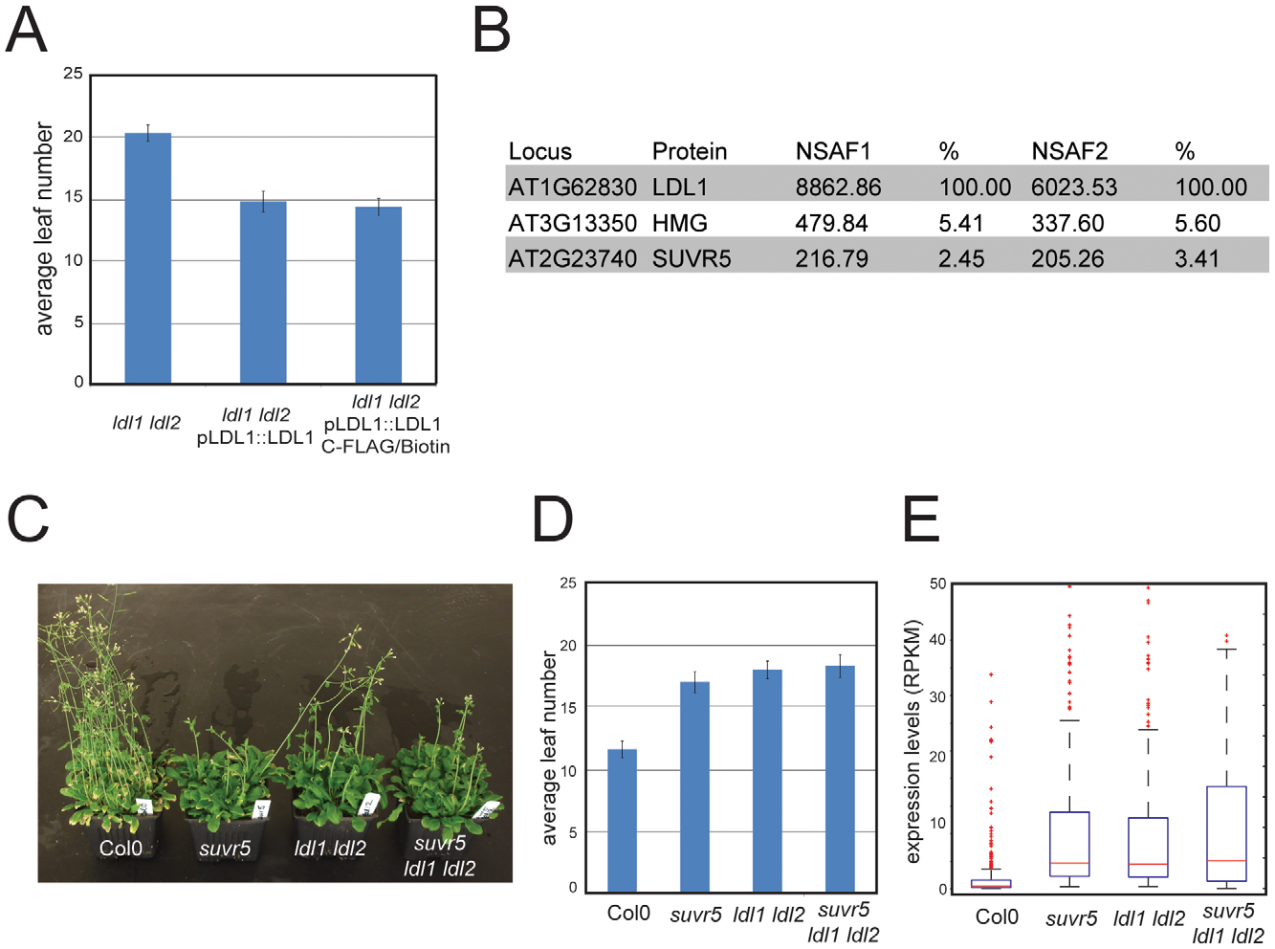
SUVR5 and LDL1 act together in a repressor complex. a, analysis of the late flowering phenotype of *ldl1 ldl2* mutants and its complementation by the tagged LDL1 transgene measured by scoring number of leaves at bolting; b, table showing the mass spectrometry analyses of LDL1 affinity purifications; c, picture showing the late flowering phenotype of *suvr5, ldl1 ldl2* and *suvr5 ldl1 ldl2* plants; d, analysis of the late flowering phenotype by scoring number of leaves at bolting; e, box plot showing the expression level (in RPKM) of the 270 genes upregulated in *suvr5* and *ldl1 ldl2* (over 4 fold and P<0.01 for both, *suvr5*/Col-0 and *ldl1 ldl2*/Col-0) in Col-0, *suvr5, ldl1 ldl2* and the triple *suvr5 ldl1 ldl2* mutants, showing the epistatic relationship between the mutants.

The majority of chromatin modifiers characterized in higher organisms are present in large multi-protein complexes. SUVR5 was shown to interact *in vitro* with the Arabidopsis homolog of LYSINE-SPECIFIC DEMETHYLASE (LSD), termed LSD-LIKE 1 (LDL1) [23], an H3K4 demethylase partially redundant with its paralog LDL2 [23]. We tested for the existence of this complex *in vivo* by generating a transgenic line that expressed a FLAG tagged version of LDL1 under its own promoter, which was shown to complement the late flowering phenotype of the *ldl1 ldl2* mutant (Figure 5a). Using affinity purification coupled with mass spectrometry (IP-Mass Spec [19]) (Figure 5b) we indeed identified an *in vivo* complex including both SUVR5 and LDL1. We also generated plants carrying a tagged version of SUVR5 expressed under the control of its own promoter, however the very poor expression levels of the tagged protein rendered our purification attempts unsuccessful.

The physical interaction between SUVR5 and LDL1 suggests that their H3K9 methyl transferase and H3K4 demethylase activities may work together in collaboration to repress gene expression. To analyze the genetic interaction between SUVR5 and LDL1 we generated the *suvr5 ldl1 ldl2* triple mutant and analyzed the effect on flowering time. Flowering time was as late in the triple mutant as in the single *suvr5* or double *ldl1 ldl2* mutants, indicating an epistatic relationship between *SUVR5* and *LDL* (Figure 5c and 5d). mRNA-Seq in the double and triple mutants revealed 270 genes that were affected by both *suvr5* and by *ldl1 ldl2* mutations, which is more than 30% of the genes controlled by *suvr5* alone. This suggests that SUVR5 and LDLs share a broad regulatory function. Furthermore, the GO category “response to stimulus” was also the most significantly enriched in *ldl1 ldl2* mutants when analyzing their upregulated genes, supporting the idea that LDL1 and SUVR5 co-regulate a diverse set of targets involved in environmental responses (for the list of genes, see Table S3, for GO term analysis, see Figure S18).

The 270 genes co-regulated by SUVR5 and LDL1 had very low expression levels in wild-type Col-0, and their degree of upregulation in the triple *suvr5 ldl1 ldl2* mutant was the same as in the single *suvr5* or double *ldl1 ldl2* mutants (Figure 5e). This confirms that the relationship between the genes is indeed epistatic, with likely their H3K9 methylation and H3K4 demethylation activities acting together to repress gene expression for a large number of genes with common biological functions. Consistent with this, the most significantly over-represented GO term for the common 270 genes was again “response to stimulus”, which supports a common role for SUVR5 and LDLs in environmental adaptation (for the list of genes, see Table S3, for GO term analysis, see Figure S19).

## Discussion

**Figure 6:**
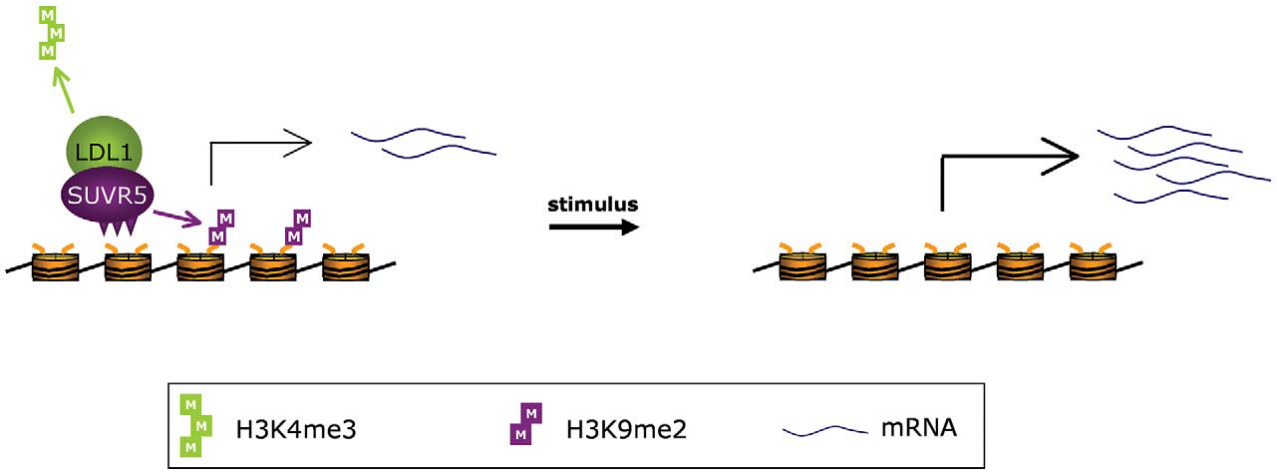
Proposed model for SUVR5 function. SUVR5 is part of a multimeric complex including LDL1 that recognizes gene promoters and represses their expression by altering their epigenetic status.

The ability of eukaryotic cells to respond to external stimuli and adapt to their environment depends on the coordinated activation and repression of specific subsets of genes. In order to facilitate this, repressive and permissive chromatin states must be readily altered in response to those stimuli. Our data are consistent with a model in which SUVR5 is part of a multimeric complex including LDL1 (and perhaps also other chromatin modifying enzymes) that recognizes genes with the sequence TACTAGTA (or related sequences) in their promoters and, in the absence of stimuli, represses their expression by altering epigenetic histone marks. This represents a unique form of epigenetic control via H3K9me2 that is independent from DNA methylation, and not perpetuated by the KYP/CMT3 loop, which potentially makes it more adaptable and dynamic for responding to environmental changes (Figure 6). One possibility is that SUVR5 mediated repression acts to modulate responses to various environmental signals as well as to provide an epigenetic memory of transcriptional states.

The functioning of SUVR5 has analogies with some repressive chromatin modifiers characterized in animals that are also present in large multiprotein complexes. One example is the mammalian silencing transcription factor REST that is important in neural differentiation. It binds to the conserved RE1 motif through its 8 Krüppel zinc finger motifs and represses many neuronal genes in non-neuronal cells [31]. This transcriptional regulation is achieved by the recruitment by REST of histone deacetylases (like HDAC1/2) [32], [33], [34], [35], demethylases (like LSD1) [36], and methyltransferases (like G9a) [37], in a similar way to the proposed SUVR5 mode of action [30]. Another example is that of PR proteins. PR (PRDI-BF1 and RIZ homology) domain proteins (PRDMs) represent a distinct and unique branch of metazoan proteins that contain a PR domain, which at the amino acid level is 20–30% identical to the SET domain found in many histone lysine methyltransferases (HMTs) [38]. The PR domain is not present in fungi or plant genomes having originated in invertebrates [39], and is almost always accompanied by C2H2-like zinc finger motifs. PRDMs act as specific transcriptional regulators catalyzing histone methylation and/or recruiting interaction partners to modify the epigenetic regulation of target genes [38]. A common feature of PRDM proteins is their ability to act as transcriptional repressors by binding both to G9a and class I histone deacetylase enzymes such as HDAC1–3 [38]. In conclusion, multisubunit complexes containing different histone modifying enzymes targeted by specific DNA binding proteins appears to be a phenomenon conserved in plant and animals and may play a greater role in gene regulation than previously appreciated.

## Materials and Methods

### Plant strains

The wild-type control in this study was the Columbia 0 ecotype (Col-0). *suvr5-1* [23] and *suvr5-2* are T-DNA insertion lines obtained from the SALK Institute Genomic Analysis Laboratory (SALK_026224 and SALK_085717 respectively). The *kyp suvh5 suvh6* line was described in [40]. The *ldl1–2 ldl2* line was described in [41].

### Recombinant protein purification

The GST fusion protein used for SELEX and EMSA experiments was made by cloning the SUVR5 zinc finger domain (aminoacids 720 to 866) using the Gateway cloning system with pDEST15 as the final destination vector. For the SAM binding assay, the SET domain was cloned (aminoacids 1078 to 1376) also in pDEST15. Protein expression and purification was performed as previously described [11] plus the addition of 100 µM ZnSO_4_ to the cell culture at the time of protein expression induction (in the case of the Zinc finger domain) and avoiding the use of EDTA during the protein purification.

### SELEX

The basic protocol for SELEX experiments described in [42] was followed with some minor modifications. For details, see Text S1. Sequencing data for the genomic SELEX experiment have been deposited at Gene Expression Omnibus (GEO) (accession number GSE39405).

### EMSA

The protocol described in [11] was followed with slight modifications to the binding buffer composition (12% glycerol, 20 mM Tris-HCl pH7.5, 50 mM KCl, 1 mM MgCl_2_, 1 mM DTT). For info on the primers used to test the protein binding, see Text S1.

### ChIP

H3K9me2 ChIP experiments were performed using 3 week old leaves of wild type Col-0 and *suvr5-1* plants, as previously described [43].

The ChIP-chip was performed as described in [20], the results show a comparison of the abundance of DNA pulled down with the anti-H3K9me2 antibody (#1220, monoclonal anti-H3K9m2 antibody, Abcam) versus INPUT.

For info on the primers used to validate the ChIP-chip results by ChIP-qPCR, see Text S1.

### ChIP–chip analysis

Each probe in the array was normalized by taking the log2 ratio of H3K9m2 to INPUT intensities, and the scores were scaled so that the average score across the arrays were zero. H3K9me2 hypomethylated regions were defined by tiling the genome into 500 bp bins (250 bp overlap), and computing the log2 ratios of the scores of *suvr5* vs Col-0, and Z-score transformed. A Z<−3 cutoff was applied, and regions within 2.5 kb were merged. Data have been deposited at Gene Expression Omnibus (GEO) (accession number GSE39405).

### Bisulfite treatment

DNA from leaves of 3 week old plants was extracted using a standard CTAB protocol. We performed sodium bisulfite treatment using EZ DNA Methylation Gold (Zymo Research) following the manufacturer's instructions, amplified specific fragments using the primers described in Text S1 and cloned the resulting PCR fragments into pCR2.1-TOPO (Invitrogen) to sequence and analyze around 20 clones per sample. To compare the converted clones to the original unconverted sequence, we used the sequence alignment tool of CLC Workbench software. We counted the converted/unconverted cytosines at each site manually and subsequently calculated the percent of methylation.

BS-Seq was performed as previously described [44]. Sequencing data have been deposited at Gene Expression Omnibus (GEO) (accession number GSE39405).

### mRNA–Seq

Leaves from wild type Col-0, *suvr5-1*, *ldl1–2 ldl2* and *suvr5-1 ldl1–2 ldl2* 3 week-old plants were used for RNA extraction using Trizol (Invitrogen) following the manufacturer instructions. 10 µg of total RNA was treated with DNaseI (Roche), and cleaned up with RNeasy columns (Qiagen). Poly(A) was purified using the Dynabeads mRNA Purification Kit (Invitrogen) and used to generate the mRNA-seq libraries following the manufacturer instructions (Illumina). The libraries were sequenced using an Illumina Genome Analyzer.

Gene and transposon expression in the RNA-seq data was measured by calculating reads per kilobase per million mapped (RPKM). P-values to detect differential expression were calculated by Fisher's exact test and Benjamini-Hochberg corrected for multiple testing. Genes differentially expressed in wild-type and mutants were defined as those that have log2(*suvr5*/wild-type)>4 and P<0.01. Sequencing data have been deposited at Gene Expression Omnibus (GEO) (accession number GSE39405).

### IP/mass spectrometry

For affinity purification of LDL1-3xFLAG ∼15 g of inflorescence tissue from transgenic and Col-0 plants was ground in liquid nitrogen, and resuspended in 75 ml of lysis buffer (50 mM Tris pH 7.5, 300 mM NaCl, 5 mM MgCl_2_, 5% glycerol v/v 0.02% NP-40 v/v, 0.5 mM DTT, 1 mg/mL pepstatin, 1 mM PMSF and 1 protease inhibitor cocktail tablet (Roche, 14696200)). Mass spectrometry analyses were performed as described in [19]. The identities of proteins co-purifying with LDL1 in Figure 5b are shown for those proteins appearing in two replicate purifications, and present at levels equivalent to at least 1% of the level of LDL1.

### Auxin treatment

Wild type Col-0, *suvr5-1* and *suvr5-2* plants were either grown for 13 days in vertical MS plates (CONTROL) or grown in vertical MS plates for 5 days before being transferred to MS+0.5 µM NAA (Sigma) plates for 7 additional days.

### GO term analysis

The web-based tool agriGO was used for the gene ontology analysis [45].

### Accession number

SUVR5 information is available in The Arabidopsis Information Resource under accession number AT2G23740.

## Supporting Information

Figure S1 *suvr5* mutants are late flowering. a, picture showing the late flowering phenotype of *suvr5-1* and *suvr1 suvr2 suvr3 suvr4 suvr5* mutants.
(TIF)

Figure S2 SUVR5 is conserved in all plant species, including moss, but not algae. ClustalW alignment of SUVR5 from *Arabidopsis thaliana* and other plant species where a homolog could be found.
(TIF)

Figure S3 Scheme explaining the SELEX experiment procedure (ss: salmon sperm DNA).
(TIF)

Figure S4 Sequencing results obtained from the SELEX experiment.
(TIF)

Figure S5 Scheme explaining the genomic SELEX experiment procedure.
(TIF)

Figure S6 SUVR5 zinc fingers binding is specific. Mobility shift assay is shown using cold competitor (250×).
(TIF)

Figure S7 SUVR5 SET domain binds SAM. SAM binding assay showing SUVR5 SET domain binds the methyl group donor S-adenosyl-l-[methyl-3H]methionine and that this interaction is avoided upon mutation of the catalytic residue 1307 from H to L (the recombinant SET domain of KYP was used as a positive control).
(TIF)

Figure S8 *suvr5* mutants show a decrease of H3K9me2 accumulation in pericentromeric heterochromatin. Chromosomal views of the log2 ratio of H3K9me2 signal in *suvr5* mutants vs. Col-0 (red), and the log2 ratio of *kyp suvh5 suvh6* triple mutants vs. Col-0 (black).
(TIF)

Figure S9 SUVR5 H3K9me2 deposition is independent of DNA methylation. a, Chromosome-wide distribution of DNA methylation in *suvr5-1* and Col-0 3-week-old rosette leaves (green = CG, blue = CHG, red = CHH; the lighter colors are Col-0, and dark colors are *suvr5-1*); b, comparison of the bulk levels of DNA methylation in the five chromosomes suggesting that there is no significant difference between the levels of methylation in wild type and *suvr5* mutants.
(TIF)

Figure S10 Validation of the BS-sequencing experiments by single locus bisulfite treated DNA PCR.
(TIF)

Figure S11 Comparison between size and DNA methylation content of TEs affected in their H3K9me2 levels redundantly by *suvr5* and *kyp suvh5 suvh6* or specifically by *suvr5*.
(TIF)

Figure S12 SUVR5-specific H3K9me2 deposition correlates with its zinc finger domain binding. Box plot showing the levels of H3K9me2 in the genes that have gSELEX signal in their upstream 3 Kb region (data from the ChIP-chip replicate).
(TIF)

Figure S13 Upregulated genes in *suvr5* are mainly localized in the chromosome arms. Chromosome-wide distribution of genes upregulated over 4 fold in *suvr5* mutants.
(TIF)

Figure S14 Examples of genes that show decreased H3K9me2 levels and increased expression in *suvr5* mutants. Validation of the ChIP-chip experiments by single locus qPCR after ChIP and mRNAseq by RT-qPCR.
(TIF)

Figure S15 Characterization of the two mutant alleles used in this study, *suvr5-1* [23] and *suvr5-2*.
(TIF)

Figure S16 AgriGO chart showing the biological process GO term clustering of the genes upregulated in *suvr5-1* (*suvr5-1* vs. Col-0, over 4 fold, P<0.01). The highlighted categories correspond to the significant ones (FDR<0.01). P-values (purple) and FDR (red) are shown for each of the significant categories.
(TIF)

Figure S17 SUVR5 binding motifs in the promoters of auxin-responsive genes AT3G12830, AT5G54490 and AT5G13320. a, nucleotide frequency matrix generated by Meme during the analysis of the genomicSELEX data, b, Binding motif occurences with p-value≤0.001 in AT3G12830, AT5G54490 and AT5G13320, calculated by FIMO motif search tool (Meme suite).
(TIF)

Figure S18 AgriGO chart showing the biological process GO term clustering of the genes upregulated in *ldl1 ldl2* (*ldl1 ldl2* vs. Col-0 over 4 fold, P<0.01). The highlighted categories correspond to the significant ones (FDR<0.01). P-values (purple) and FDR (red) are shown for each of the significant categories.
(TIF)

Figure S19 AgriGO chart showing the biological process GO term clustering of the genes upregulated in both *suvr5-1* and *ldl1 ldl2* (270 genes). The highlighted categories correspond to the significant ones (FDR<0.01). P-values (purple) and FDR (red) are shown for each of the significant categories.
(TIF)

Table S1 Table showing the upregulated genes in *suvr5-1* mature leaves (over 4 fold and P<0.01).
(XLS)

Table S2 Table showing the upregulated TEs in *suvr5-1* mature leaves (over 4 fold and P<0.01).
(XLS)

Table S3 Table showing the upregulated genes in *ldl1 ldl2* mature leaves (over 4 fold and P<0.01) and the subset of those in common with *suvr5-1* (270 genes).
(XLS)

Text S1 Supplemental Materials and Methods and list of primers used.
(DOC)
