# Supplementary figures and images for: The SET-Domain Protein SUVR5 Mediates H3K9me2 Deposition and Silencing at Stimulus Response Genes in a DNA Methylation–Independent Manner
Source: PLoS Genet. 2012 Oct 11;8(10):e1002995. doi: 10.1371/journal.pgen.1002995 (PMC3469426; doi:10.1371/journal.pgen.1002995)

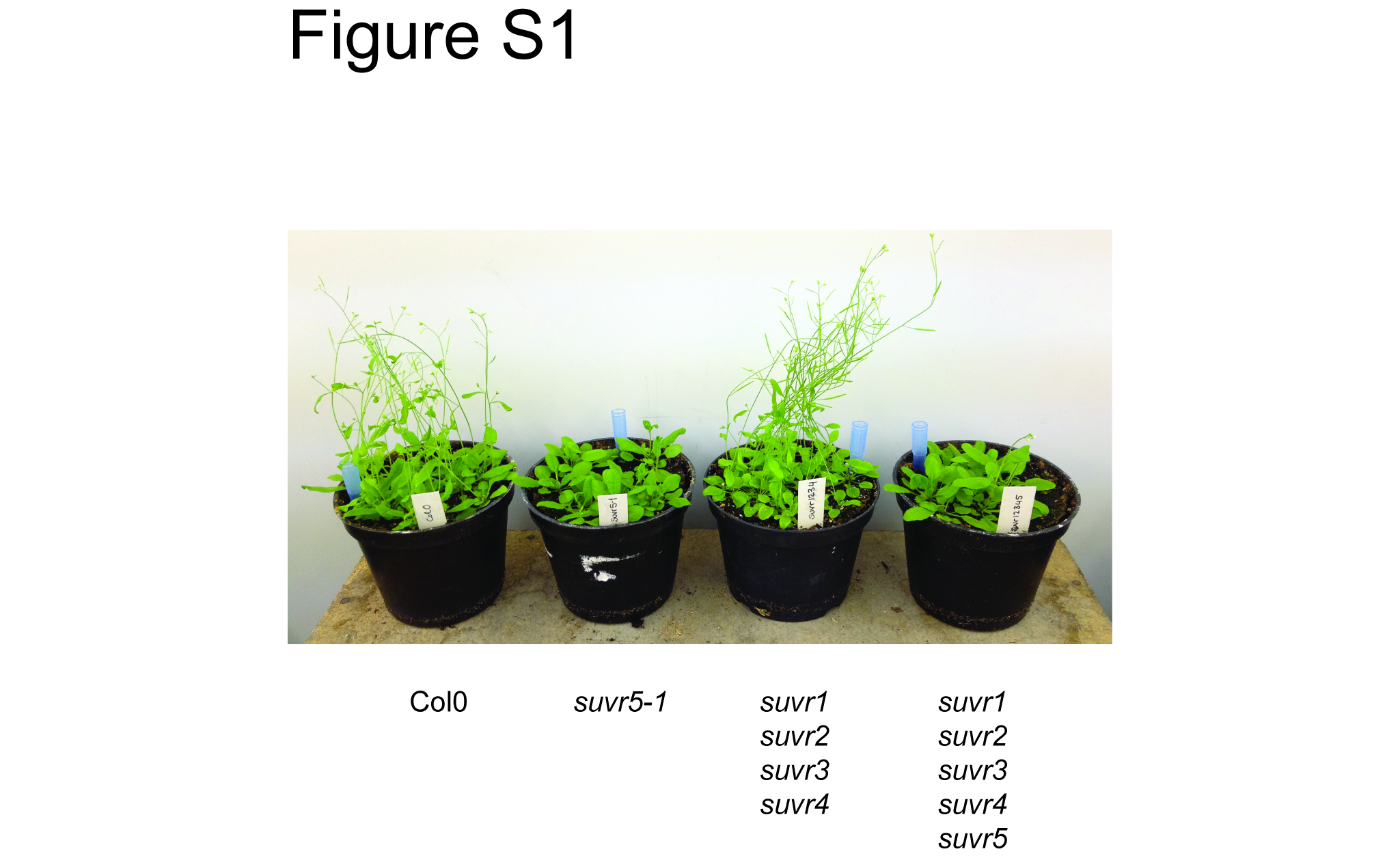

Supplement: Figure S1 — suvr5 mutants are late flowering. a, picture showing the late flowering phenotype of suvr5-1 and suvr1 suvr2 suvr3 suvr4 suvr5 mutants. (TIF) [file pgen.1002995.s001.tif]

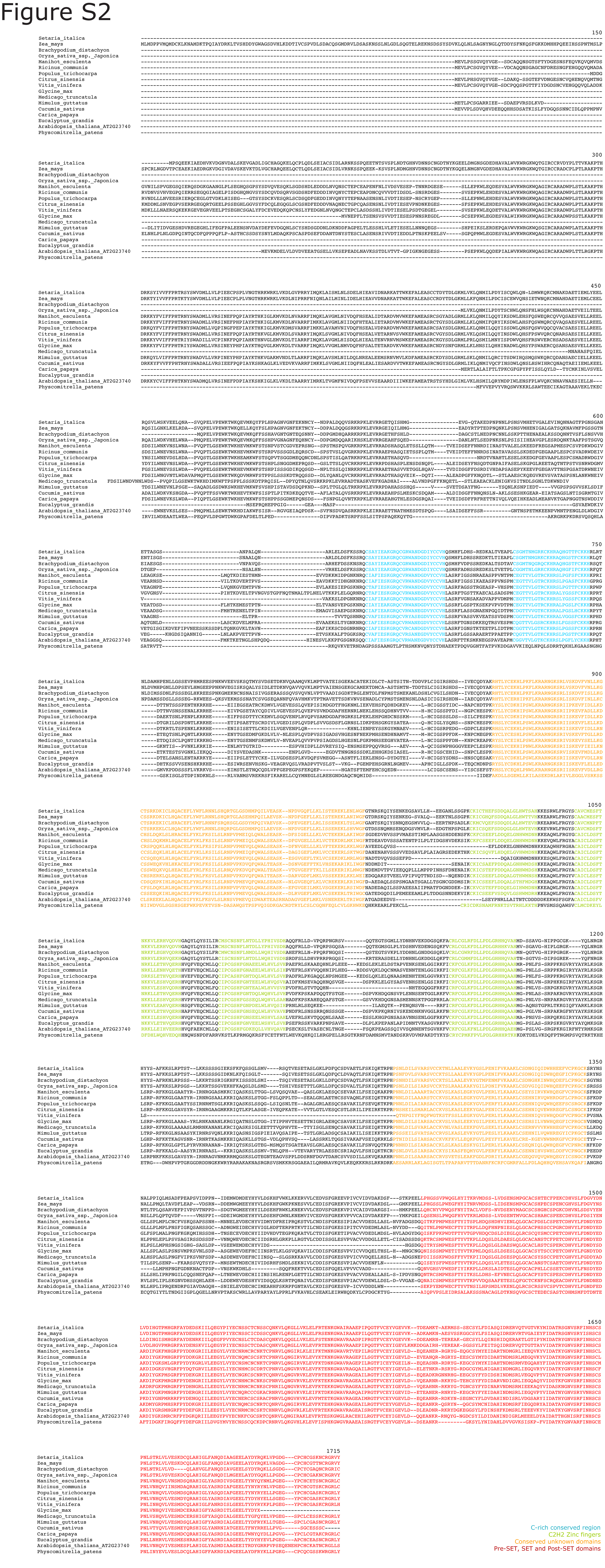

Supplement: Figure S2 — SUVR5 is conserved in all plant species, including moss, but not algae. ClustalW alignment of SUVR5 from Arabidopsis thaliana and other plant species where a homolog could be found. (TIF) [file pgen.1002995.s002.tif]

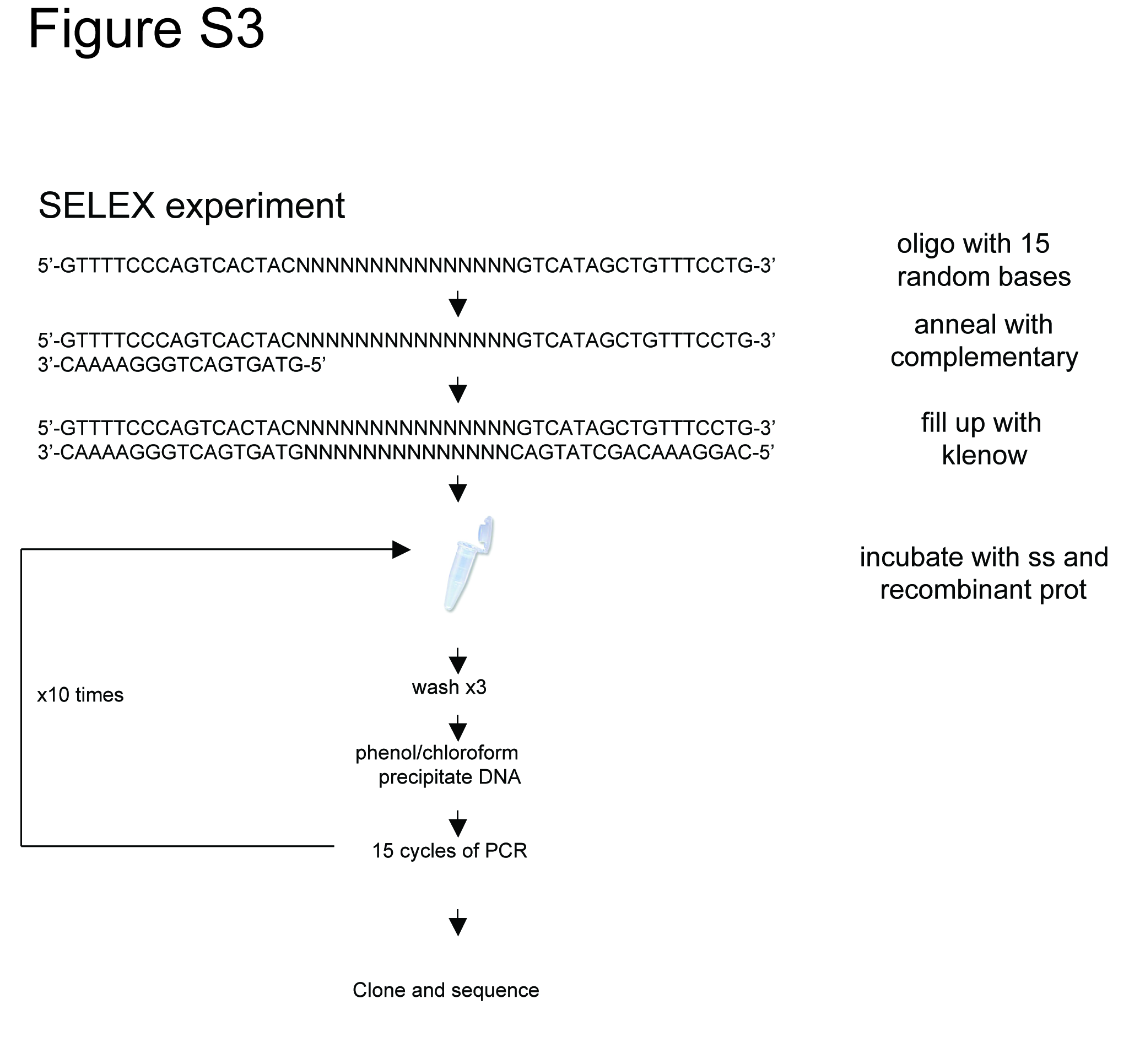

Supplement: Figure S3 — Scheme explaining the SELEX experiment procedure (ss: salmon sperm DNA). (TIF) [file pgen.1002995.s003.tif]

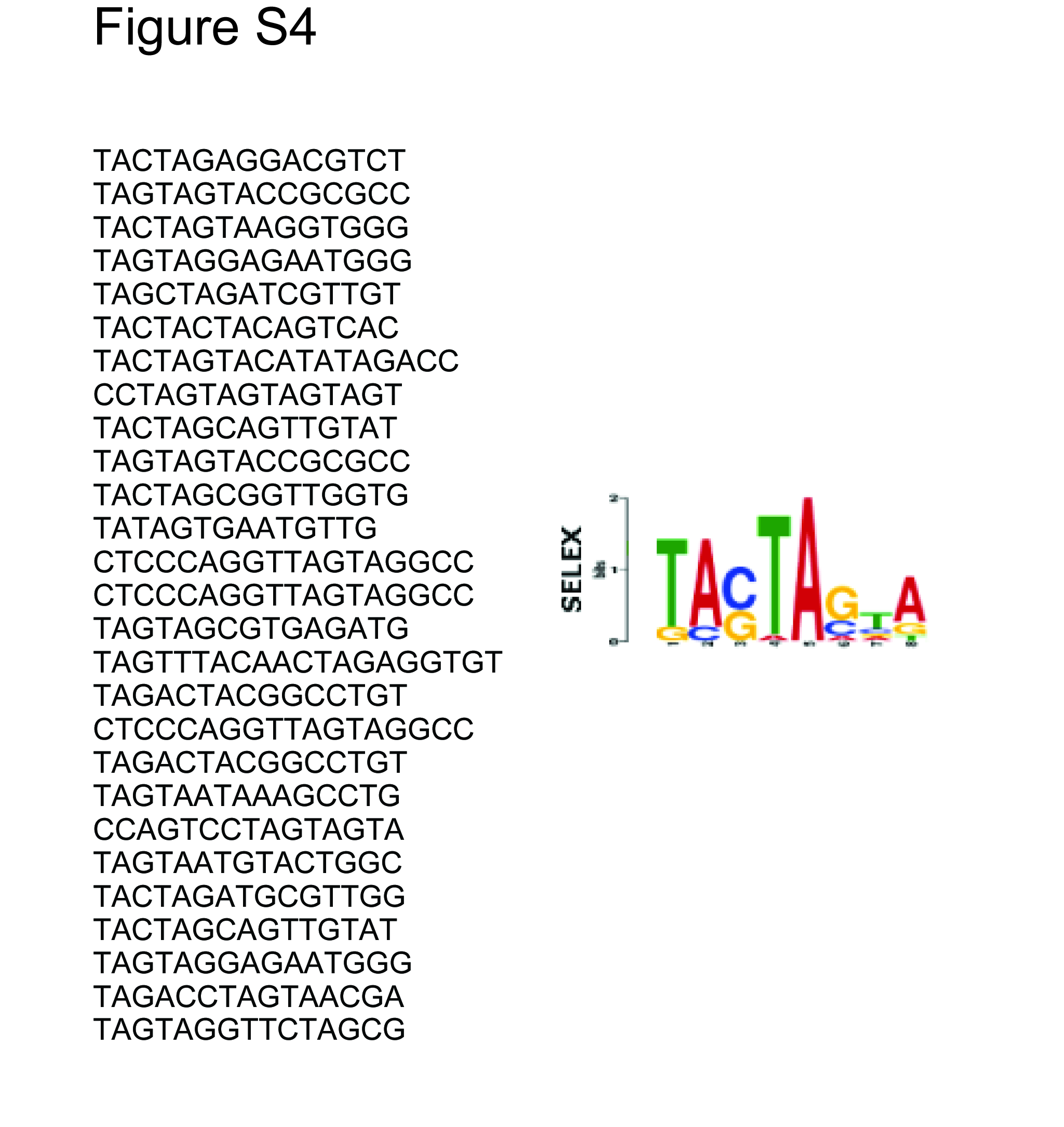

Supplement: Figure S4 — Sequencing results obtained from the SELEX experiment. (TIF) [file pgen.1002995.s004.tif]

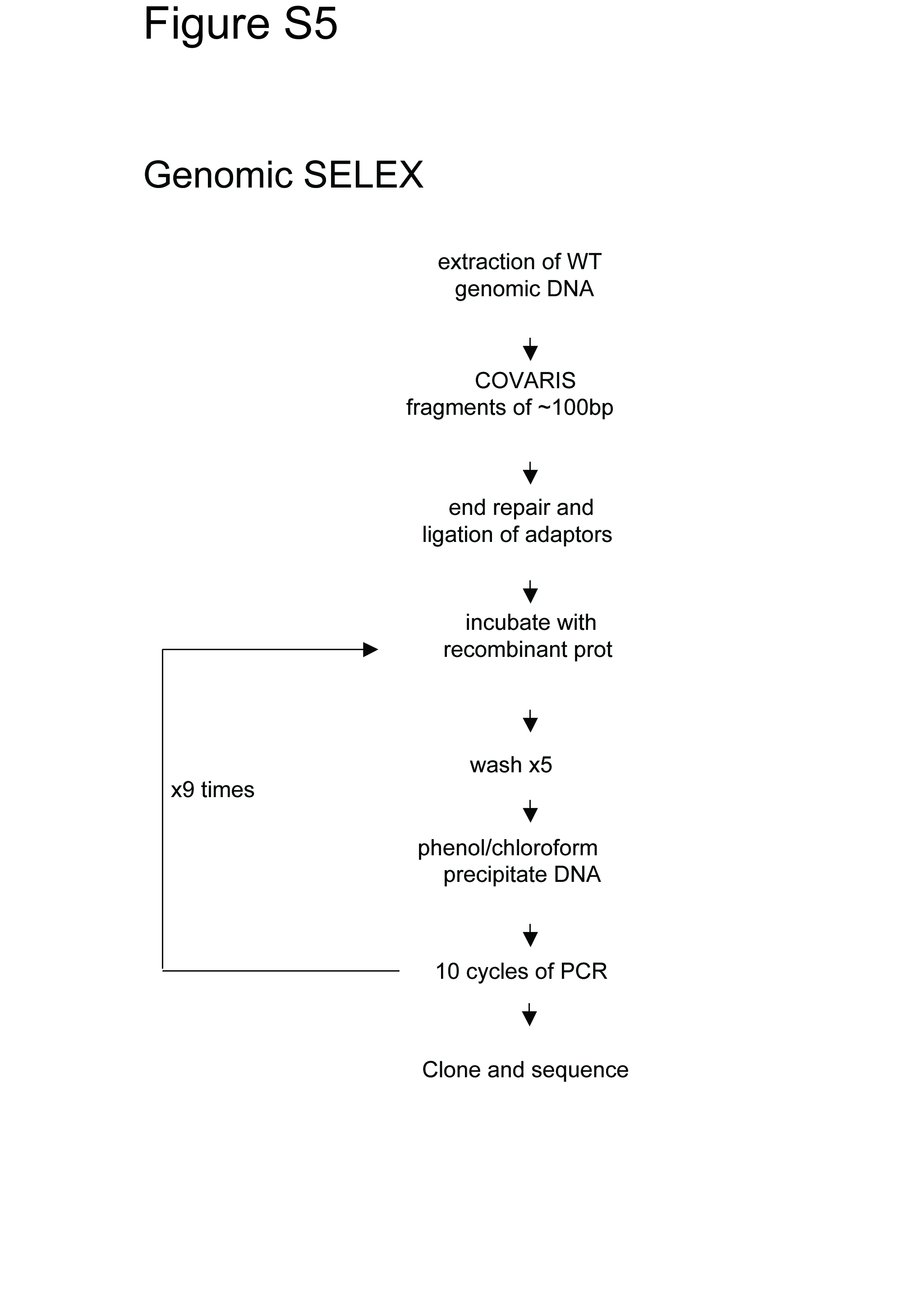

Supplement: Figure S5 — Scheme explaining the genomic SELEX experiment procedure. (TIF) [file pgen.1002995.s005.tif]

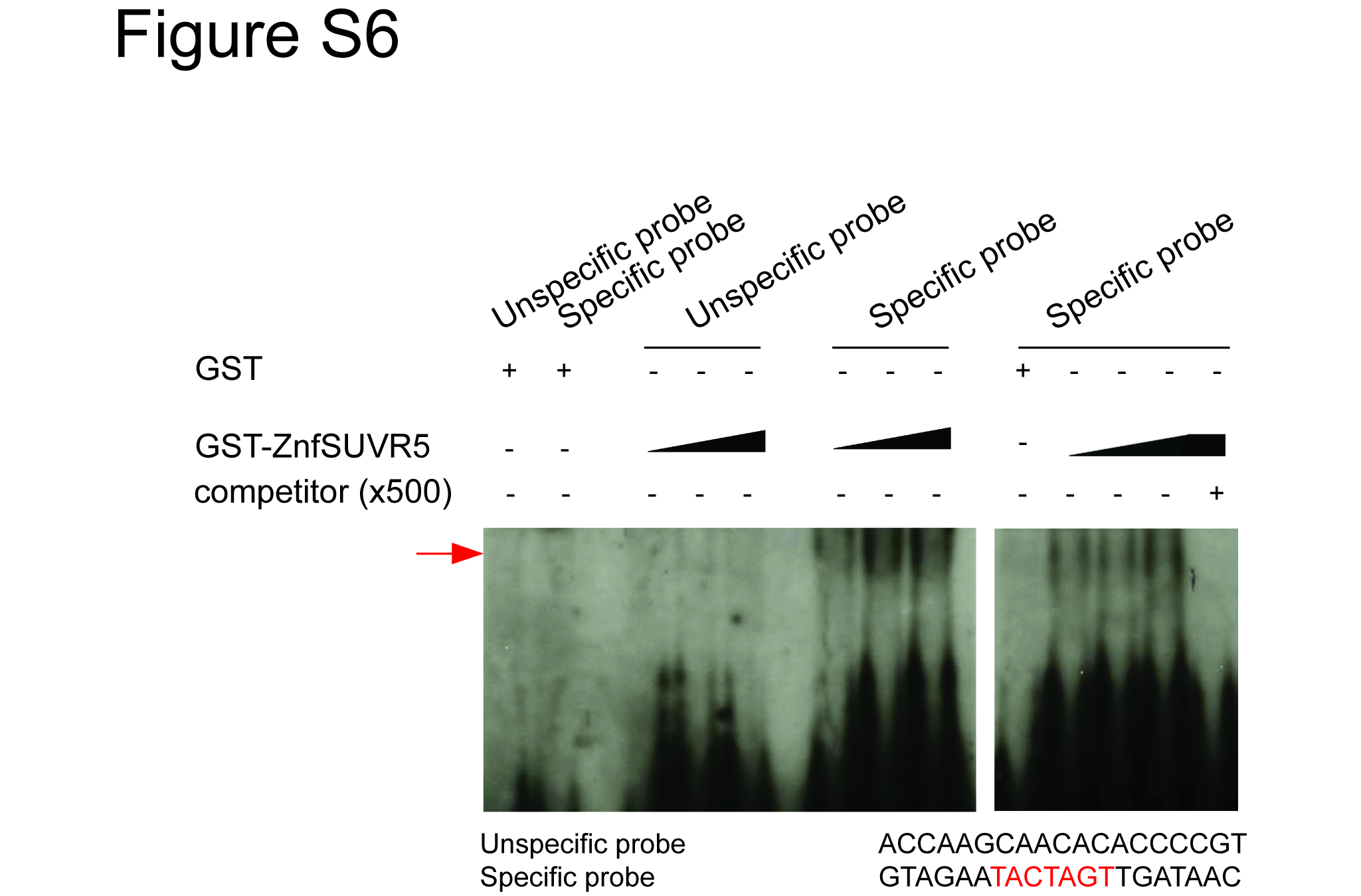

Supplement: Figure S6 — SUVR5 zinc fingers binding is specific. Mobility shift assay is shown using cold competitor (250×). (TIF) [file pgen.1002995.s006.tif]

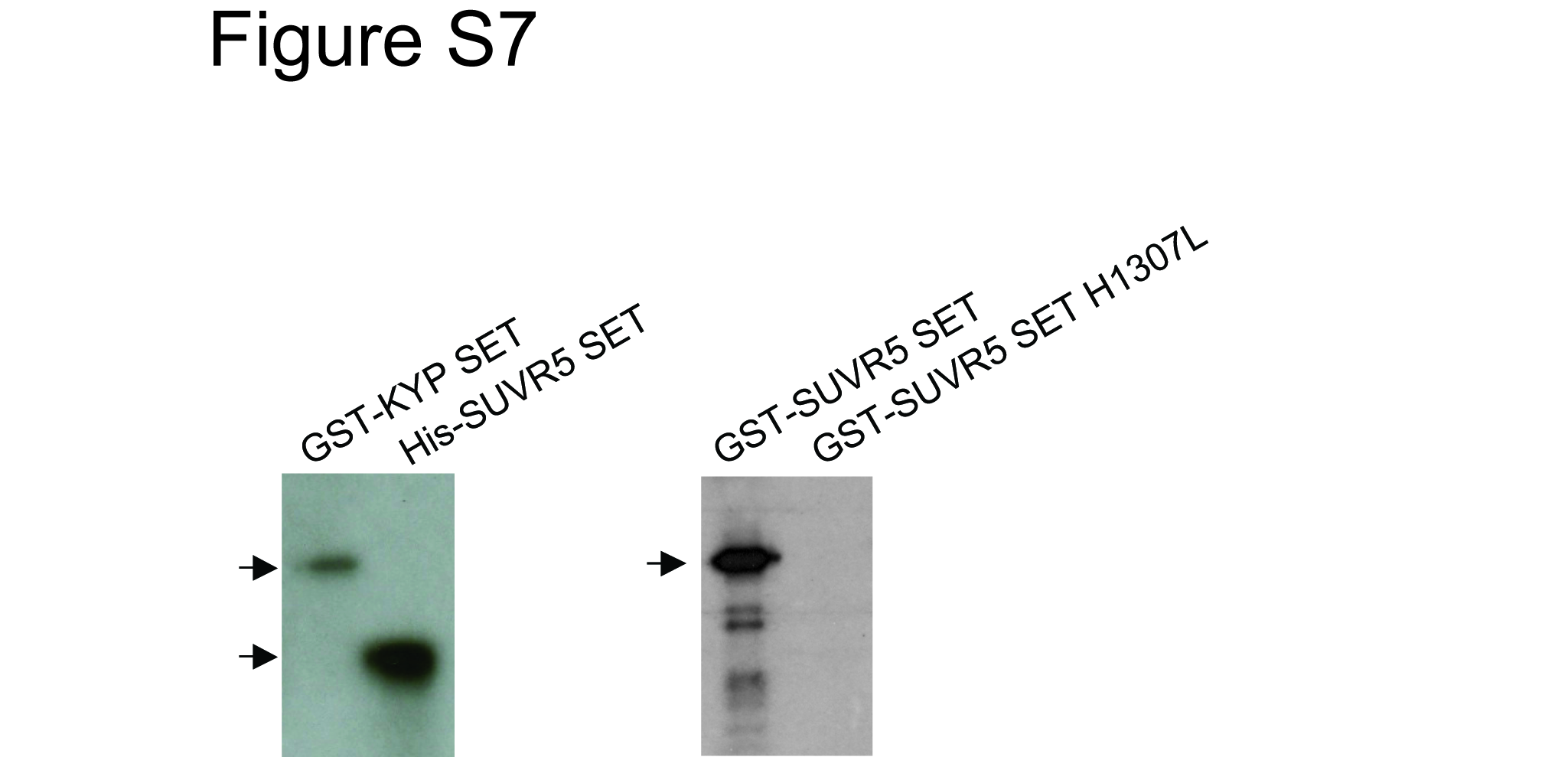

Supplement: Figure S7 — SUVR5 SET domain binds SAM. SAM binding assay showing SUVR5 SET domain binds the methyl group donor S-adenosyl-l-[methyl-3H]methionine and that this interaction is avoided upon mutation of the catalytic residue 1307 from H to L (the recombinant SET domain of KYP was used as a positive control). (TIF) [file pgen.1002995.s007.tif]

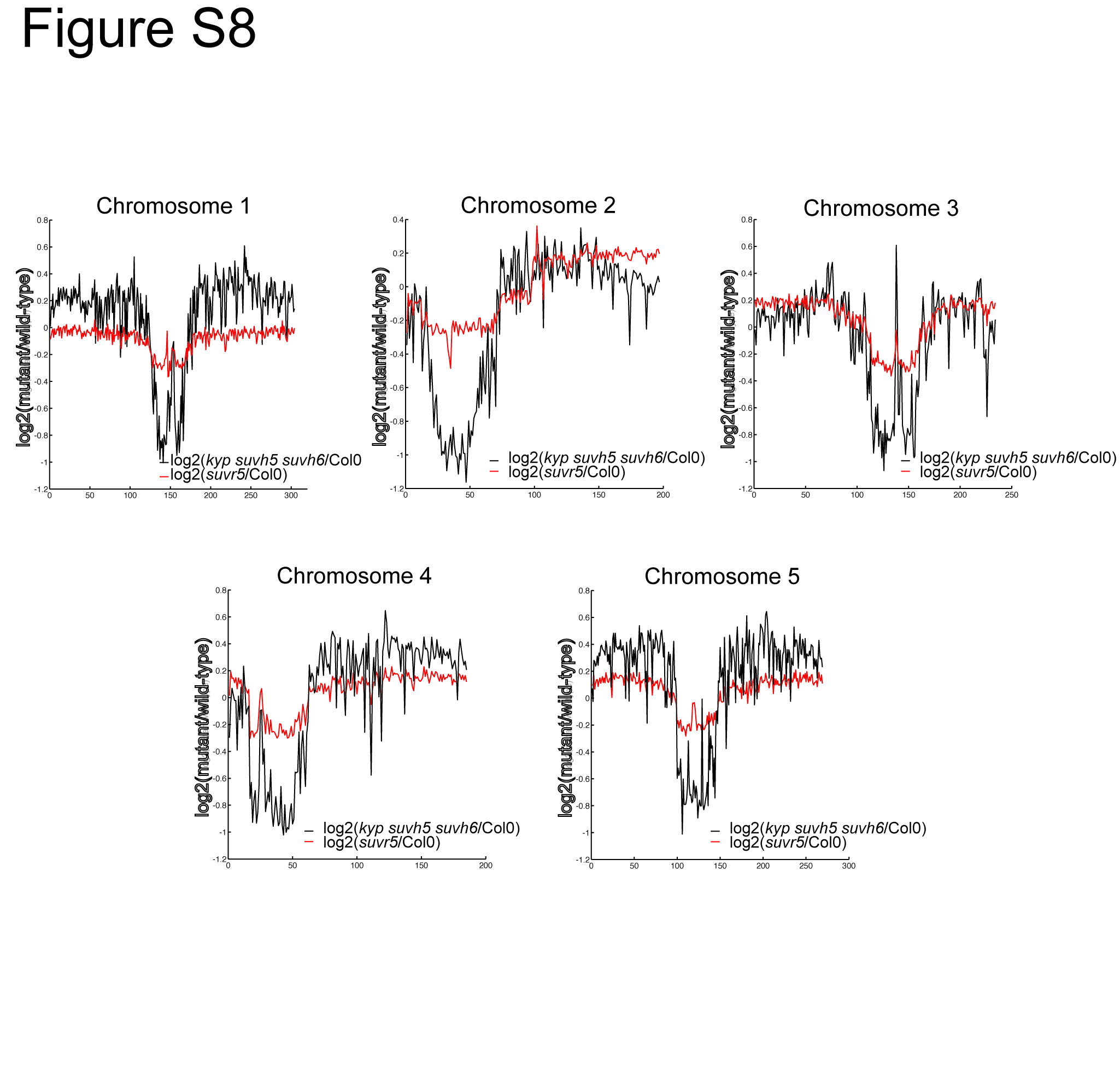

Supplement: Figure S8 — suvr5 mutants show a decrease of H3K9me2 accumulation in pericentromeric heterochromatin. Chromosomal views of the log2 ratio of H3K9me2 signal in suvr5 mutants vs. Col-0 (red), and the log2 ratio of kyp suvh5 suvh6 triple mutants vs. Col-0 (black). (TIF) [file pgen.1002995.s008.tif]

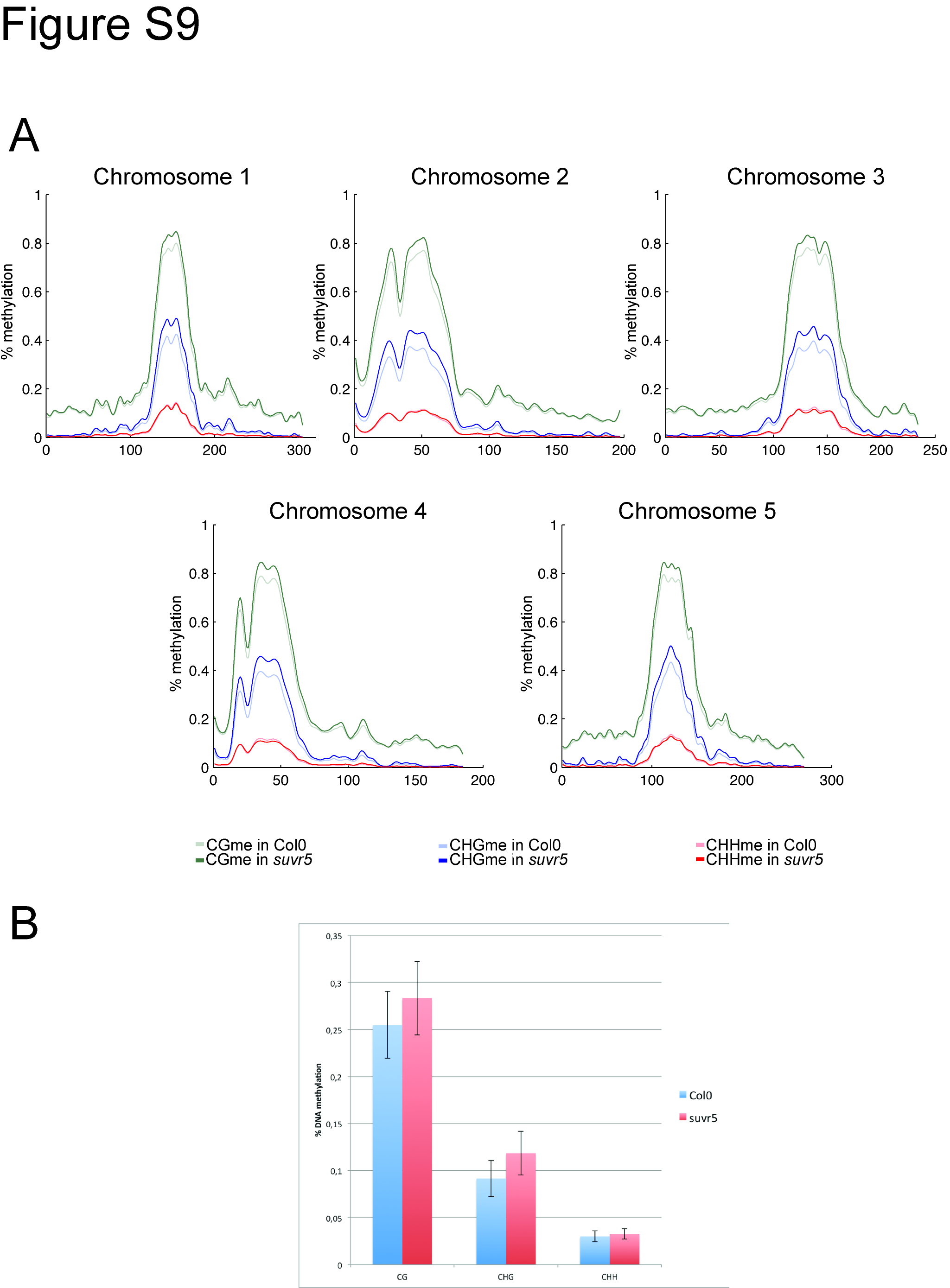

Supplement: Figure S9 — SUVR5 H3K9me2 deposition is independent of DNA methylation. a, Chromosome-wide distribution of DNA methylation in suvr5-1 and Col-0 3-week-old rosette leaves (green = CG, blue = CHG, red = CHH; the lighter colors are Col-0, and dark colors are suvr5-1); b, comparison of the bulk levels of DNA methylation in the five chromosomes suggesting that there is no significant difference between the levels of methylation in wild type and suvr5 mutants. (TIF) [file pgen.1002995.s009.tif]

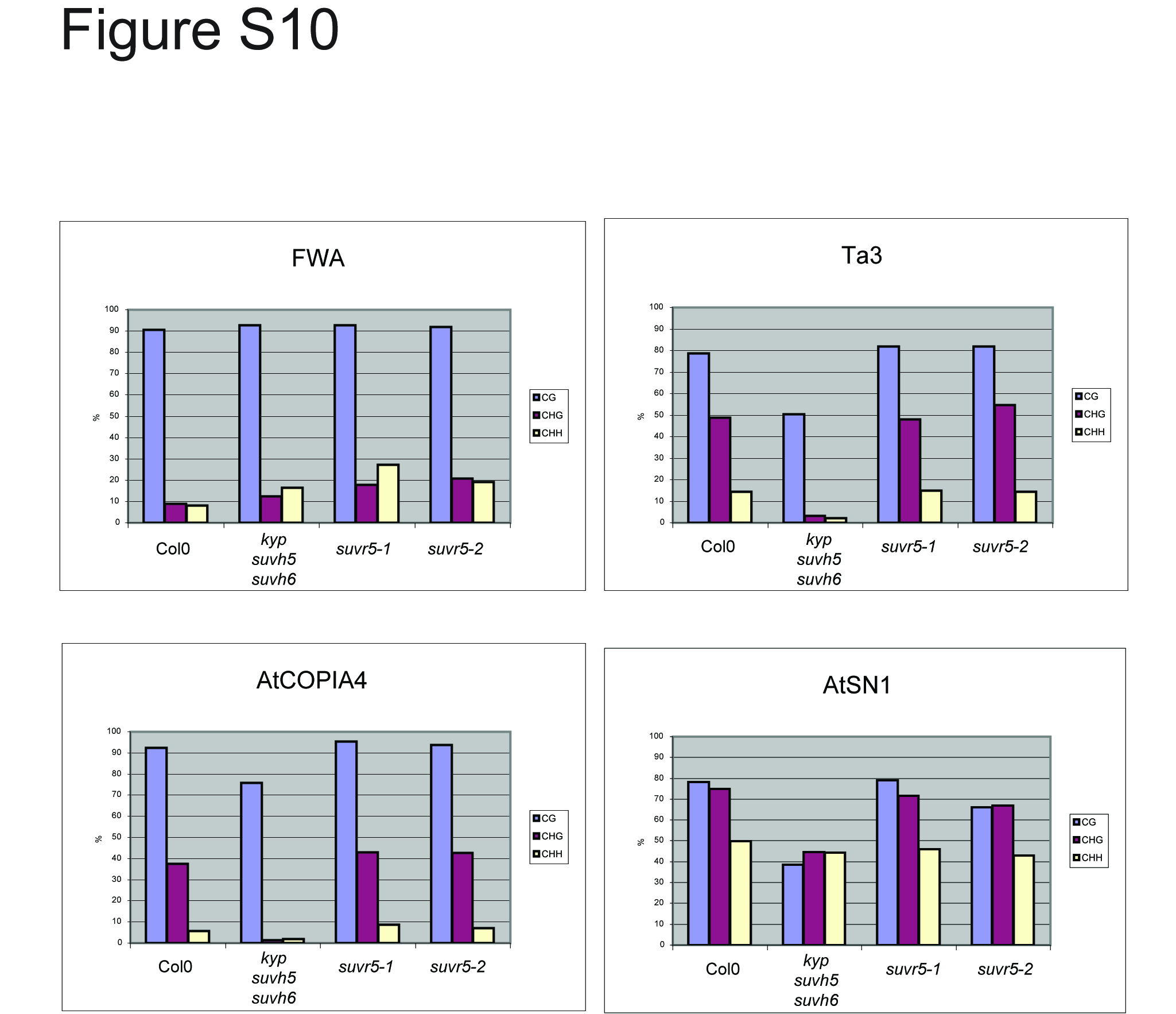

Supplement: Figure S10 — Validation of the BS-sequencing experiments by single locus bisulfite treated DNA PCR. (TIF) [file pgen.1002995.s010.tif]

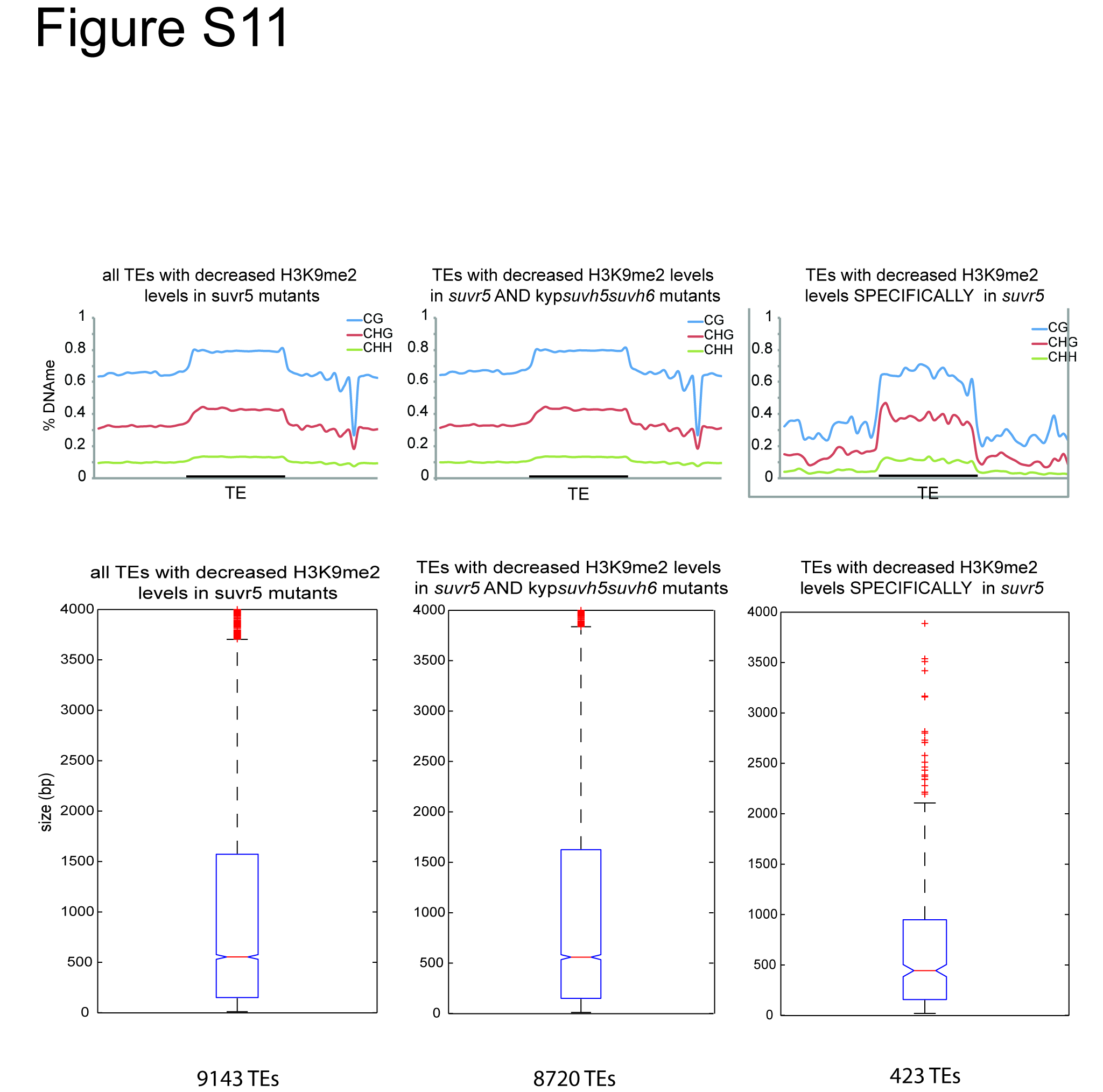

Supplement: Figure S11 — Comparison between size and DNA methylation content of TEs affected in their H3K9me2 levels redundantly by suvr5 and kyp suvh5 suvh6 or specifically by suvr5. (TIF) [file pgen.1002995.s011.tif]

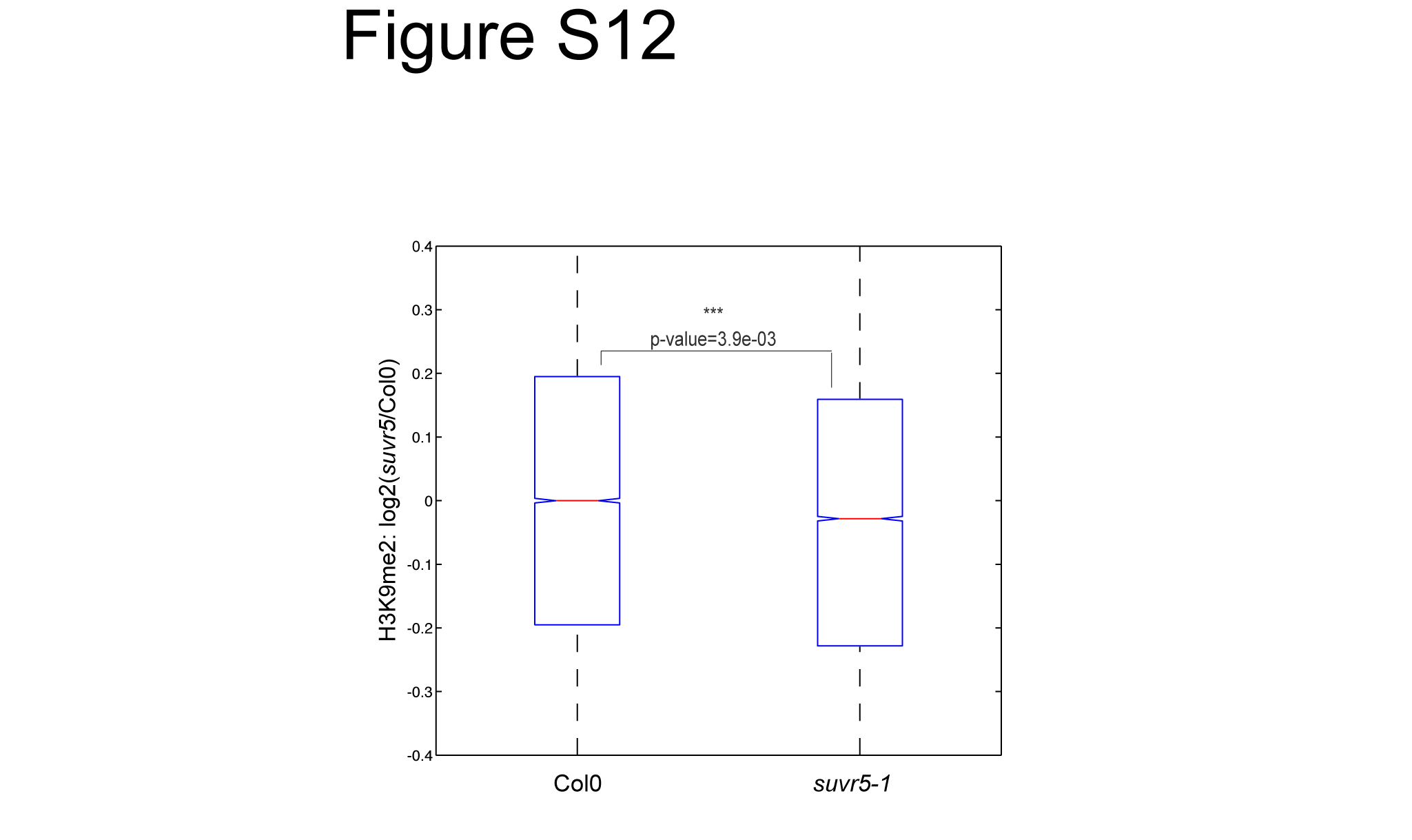

Supplement: Figure S12 — SUVR5-specific H3K9me2 deposition correlates with its zinc finger domain binding. Box plot showing the levels of H3K9me2 in the genes that have gSELEX signal in their upstream 3 Kb region (data from the ChIP-chip replicate). (TIF) [file pgen.1002995.s012.tif]

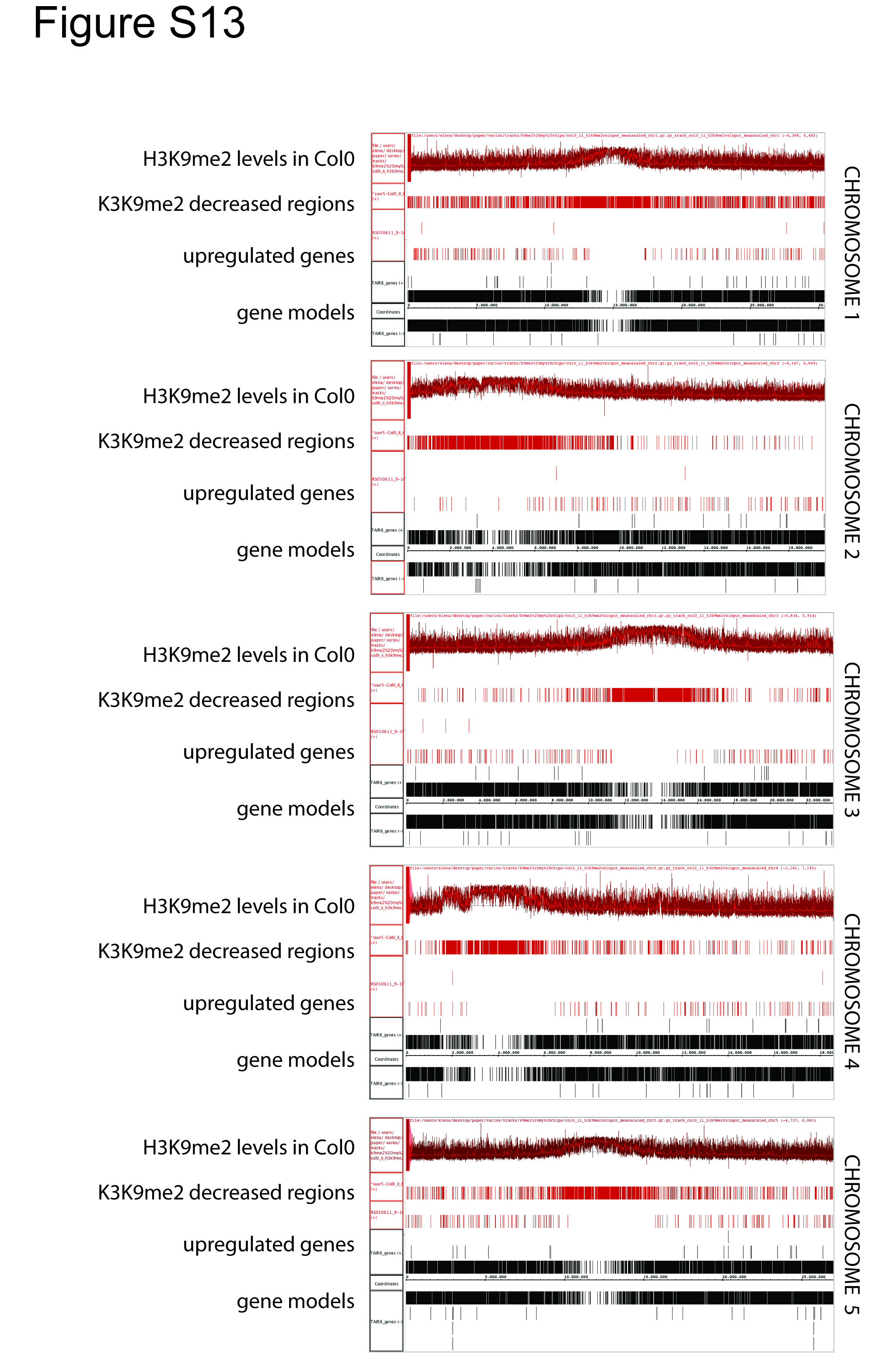

Supplement: Figure S13 — Upregulated genes in suvr5 are mainly localized in the chromosome arms. Chromosome-wide distribution of genes upregulated over 4 fold in suvr5 mutants. (TIF) [file pgen.1002995.s013.tif]

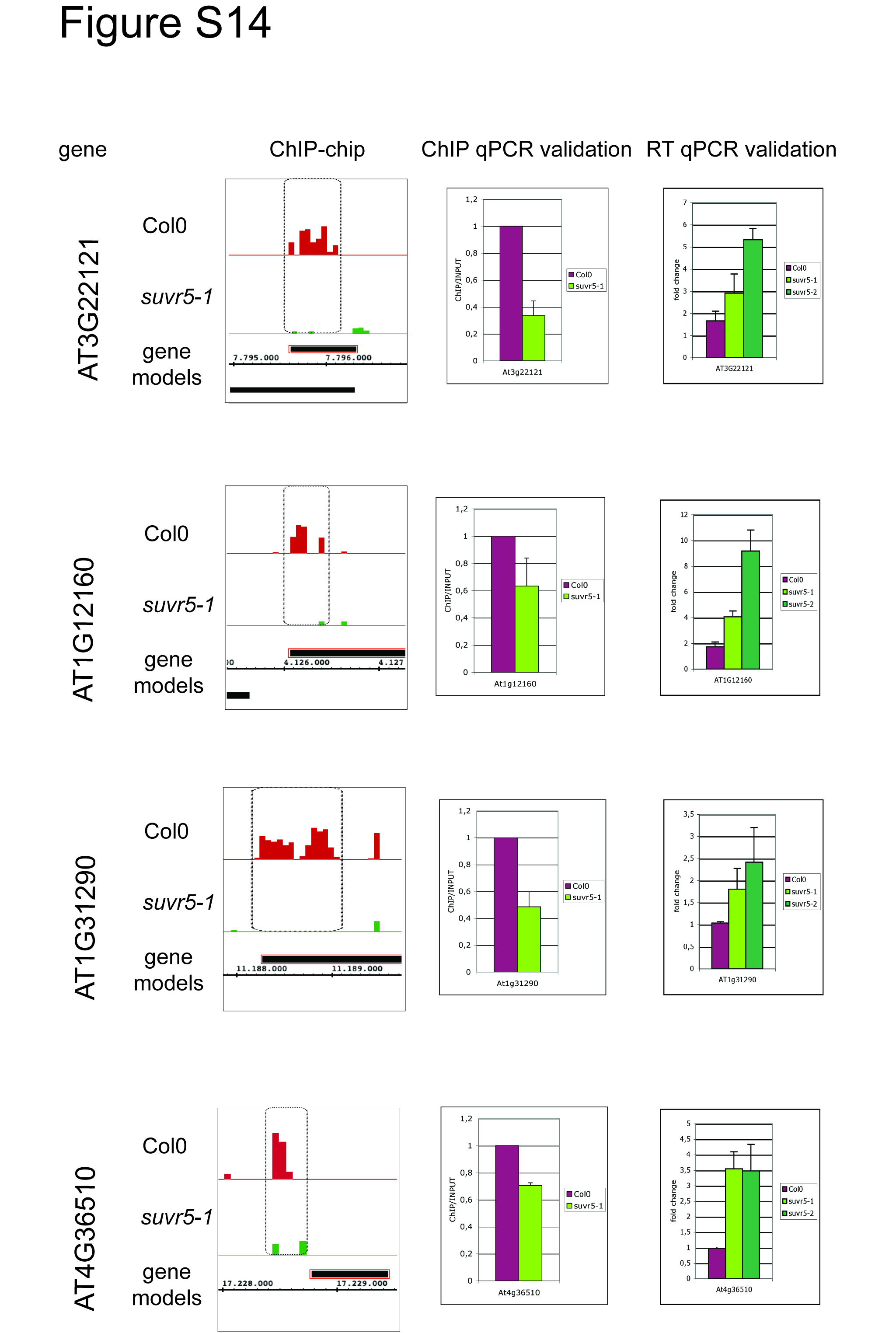

Supplement: Figure S14 — Examples of genes that show decreased H3K9me2 levels and increased expression in suvr5 mutants. Validation of the ChIP-chip experiments by single locus qPCR after ChIP and mRNAseq by RT-qPCR. (TIF) [file pgen.1002995.s014.tif]

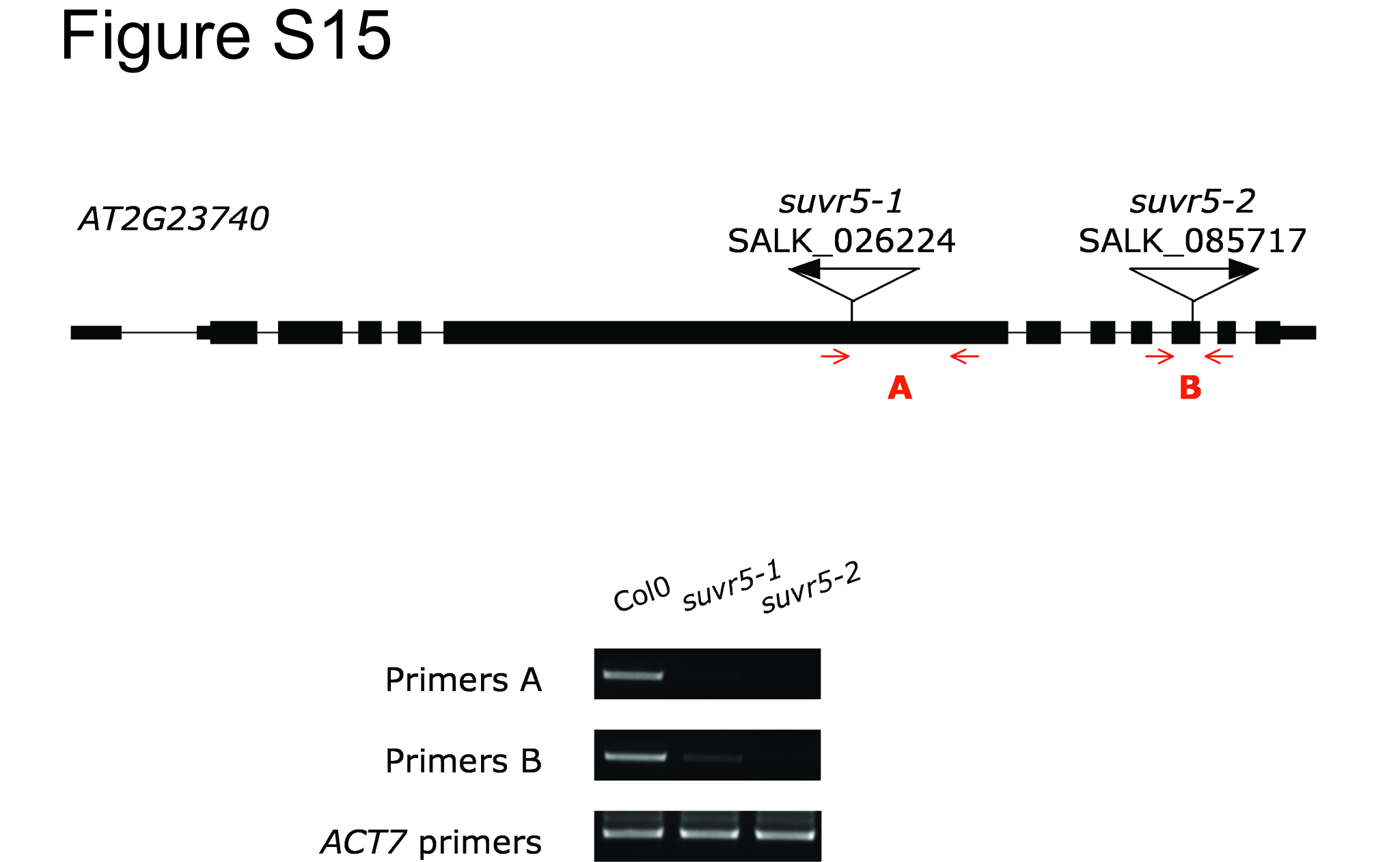

Supplement: Figure S15 — Characterization of the two mutant alleles used in this study, suvr5-1 [23] and suvr5-2. (TIF) [file pgen.1002995.s015.tif]

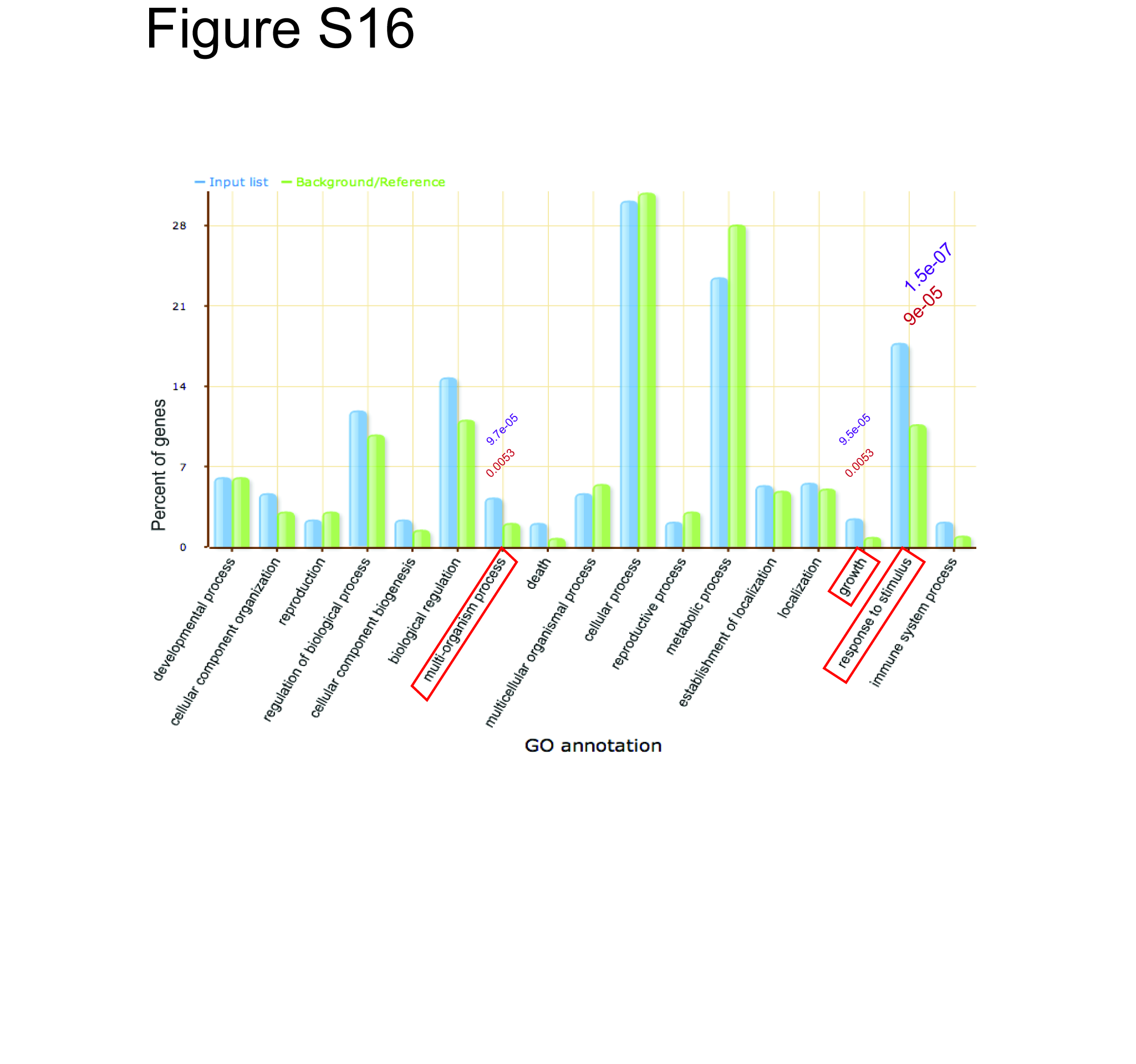

Supplement: Figure S16 — AgriGO chart showing the biological process GO term clustering of the genes upregulated in suvr5-1 (suvr5-1 vs. Col-0, over 4 fold, P<0.01). The highlighted categories correspond to the significant ones (FDR<0.01). P-values (purple) and FDR (red) are shown for each of the significant categories. (TIF) [file pgen.1002995.s016.tif]

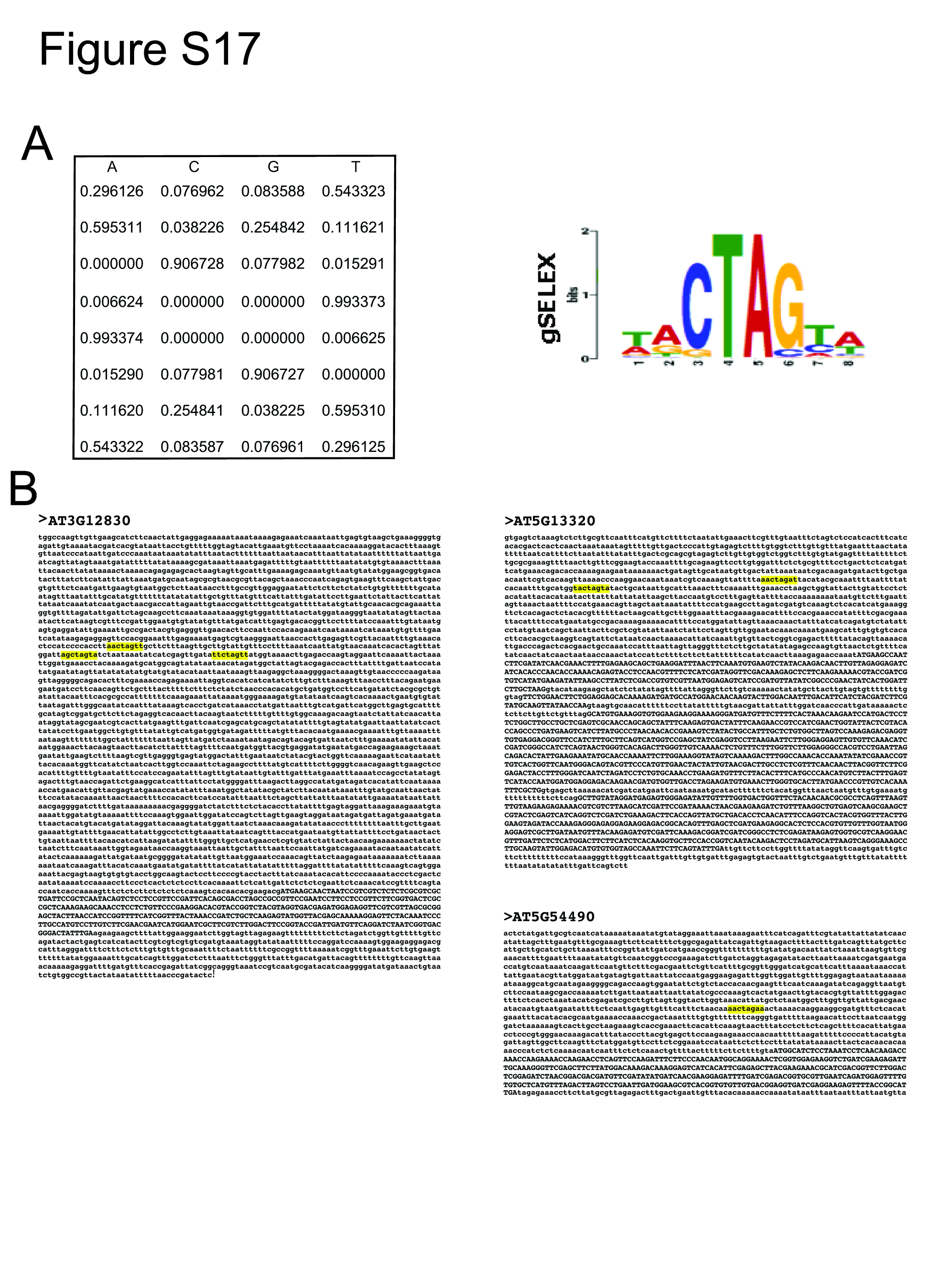

Supplement: Figure S17 — SUVR5 binding motifs in the promoters of auxin-responsive genes AT3G12830, AT5G54490 and AT5G13320. a, nucleotide frequency matrix generated by Meme during the analysis of the genomicSELEX data, b, Binding motif occurences with p-value≤0.001 in AT3G12830, AT5G54490 and AT5G13320, calculated by FIMO motif search tool (Meme suite). (TIF) [file pgen.1002995.s017.tif]

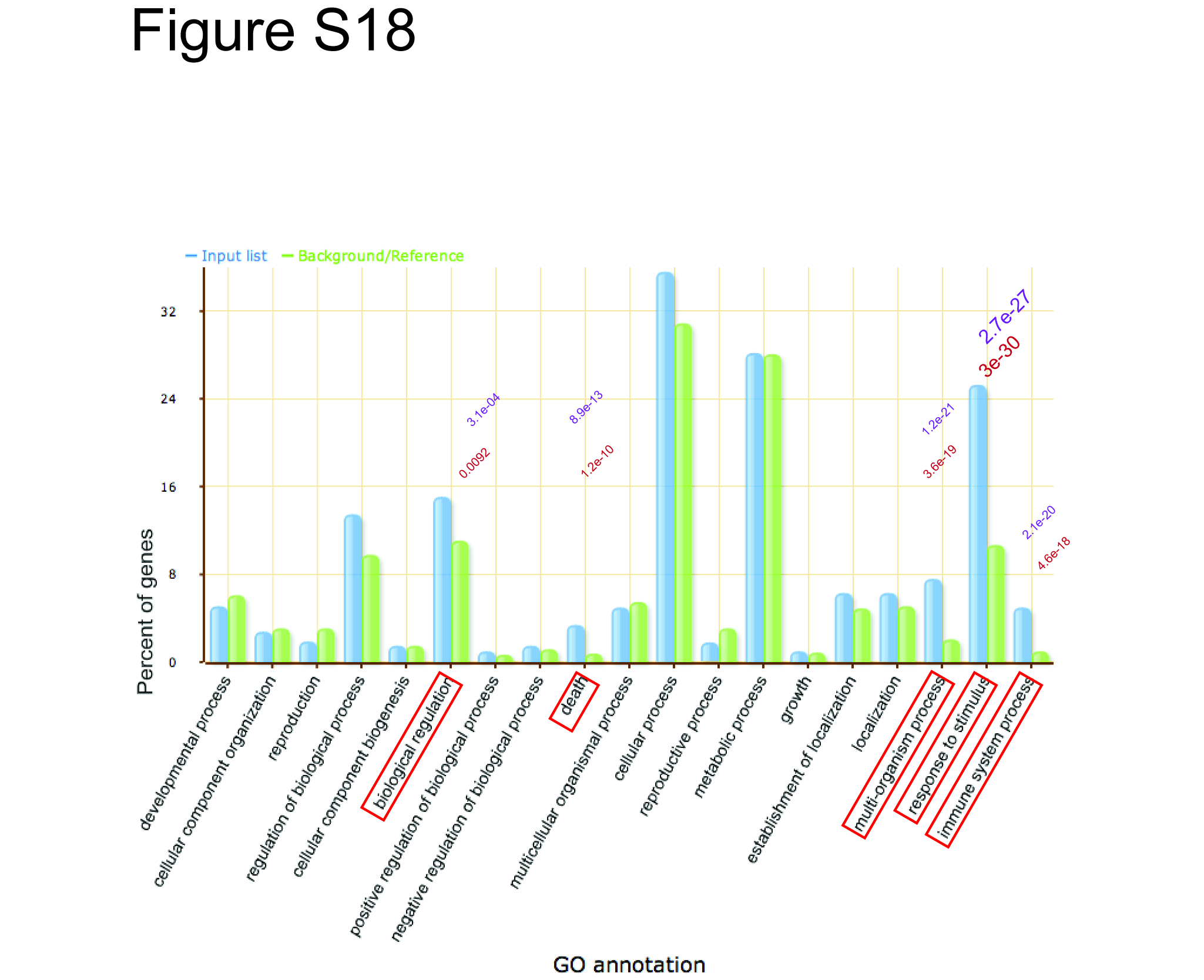

Supplement: Figure S18 — AgriGO chart showing the biological process GO term clustering of the genes upregulated in ldl1 ldl2 (ldl1 ldl2 vs. Col-0 over 4 fold, P<0.01). The highlighted categories correspond to the significant ones (FDR<0.01). P-values (purple) and FDR (red) are shown for each of the significant categories. (TIF) [file pgen.1002995.s018.tif]

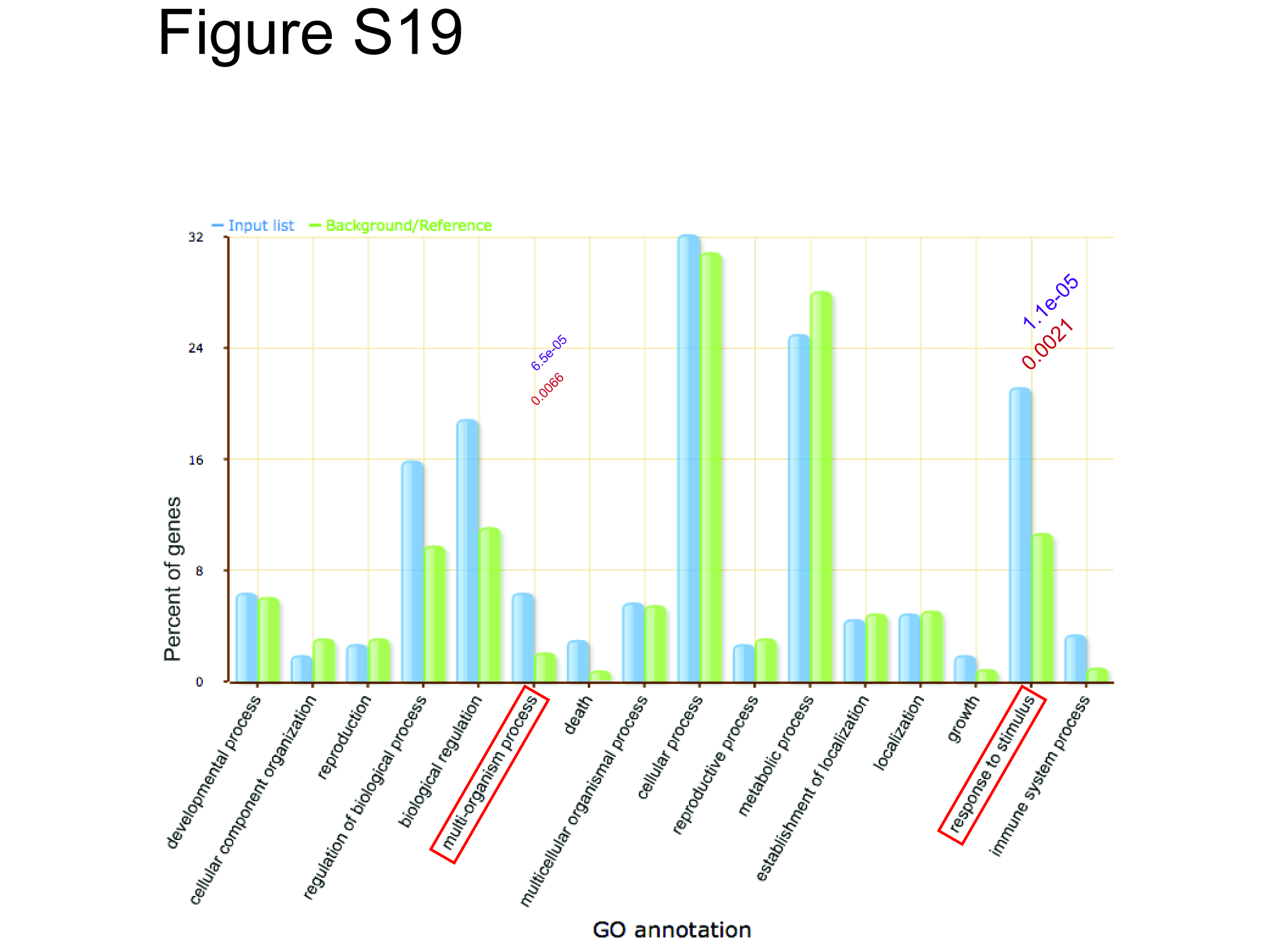

Supplement: Figure S19 — AgriGO chart showing the biological process GO term clustering of the genes upregulated in both suvr5-1 and ldl1 ldl2 (270 genes). The highlighted categories correspond to the significant ones (FDR<0.01). P-values (purple) and FDR (red) are shown for each of the significant categories. (TIF) [file pgen.1002995.s019.tif]
