# Supplementary material for: The SET-Domain Protein SUVR5 Mediates H3K9me2 Deposition and Silencing at Stimulus Response Genes in a DNA Methylation–Independent Manner
Source: PLoS Genet. 2012 Oct 11;8(10):e1002995. doi: 10.1371/journal.pgen.1002995 (PMC3469426; doi:10.1371/journal.pgen.1002995)
Supplement: Text S1 — Supplemental Materials and Methods and list of primers used. (DOC) [file pgen.1002995.s023.doc]

#### **Text S1**

#### Alignments

The identification of SUVR5 plant homologs, their sequences and their alignment was obtained from Phytozome.

#### SAM binding assay

The binding assay was performed as previously described with minor modification [41]. Specifically, proteins were incubated with 0.5 µCi of S-adenosyl-L-[methyl-3H] methionine in 30 µl reaction overnight at 4°C. They were exposed to UV in a crosslinker and the proteins were separated on SDS-PAGE and subjected to fluorography.

#### RT-qPCR

RNA was extracted from 0.2 g of tissue using Trizol (Invitrogen) and following the manufacturer’s instructions. 1 μg of total RNA was used for RT-PCR using SuperScript III (Invitrogen). qPCR was performed using iQ SYBR Green Supermix (#170-8880, BioRad). Three biological replicas were sampled and standard deviations determined. The primers used were designed using QuantPrime qPCR primer design tool, for information on their sequence, see Supporting Information.

#### SELEX

For the SELEX experiments, 5 μg of a primer with 15 random nucleotides between two adaptor sequences (JP7666: GTT TTC CCA GTC ACT ACN NNN NNN NNN NNN NNG TCA TAG CTG TTT CCT G) was annealed with 5 μg of the reverse adaptor primer JP7668 (CAGGAAACAGCTATGAC) by boiling and letting them cool down slowly. Then, 1 μg of the annealed primers was used to make dsDNA using Klenow fragment, followed by a standard phenol DNA extraction and resuspension in 200 μL of SELEX binding buffer (25mM HEPES pH7.5, 50mM KCl, 2.5mM MgCl_2_, 0.1%NP40, 1μM ZnSO_4_, 5% glycerol).

The purified and glutathione beads-bound GST-SUVR5 zinc finger domain was incubated with the dsDNA in SELEX buffer, 5μg of BSA and 5μg of salmon sperm DNA for 30 minutes at room temperature. The beads were washed 5 times with 1mL of SELEX binding buffer followed by a Phenol/Chloroform/IAA DNA extraction and precipitation. The recovered DNA was resuspended in 10 μL of TE buffer and used for PCR as follows: (95ºC for 3’), (95ºC for 30’’; 60ºC for 1’; 72ºC for 30’’)x10 cycles, (72ºC for 10’). The result of the PCR was used as a starting point for the next binding/eluting cycle.

For the standard SELEX experiment, 10 cycles of binding/eluting were done before TOPO ligating the recovered DNA to pCR2.1 vector (Invitrogen) and transforming E. coli TOP10 bacteria (Invitrogen). 20 colonies were sequenced, and the sequencing data were used to identify the consensus binding motif using the MEME Suite [43].

For the genomic SELEX experiment, Arabidopsis thaliana genomic DNA was extracted from wild type 3 week old plant leaves and fragmented to 100 bp using COVARIS. 2 μg of this DNA was processed for end repair and adaptor ligation following the manufacturer’s instructions (Illumina) and used as indicated above for incubation with purified GST-SUVR5 zinc finger domain protein. Two genomic SELEX experiments were performed, one using only one binding/eluting cycle (x1: control) and one with 9 cycles (x9). The recovered DNA was sequenced using an Illumina Genome Analyzer. gSELEX peaks were defined using MACS with the following parameters: band width = 100, model fold =32, p<1e-10, and for the definition of SELEX peaks we considered a peak positive when it showed over 50 fold increase.

A random thousand reads were used to identify a binding motif sequence using the analysis tool MEME Suite [43].

Primers used for ChIP-chip validation:

| **PRIMER NAME** | **SEQUENCE** | **GENE** |
| --- | --- | --- |
| JP2454 | TCTCTCTCGCTGCTTCTCG | ACT7 |
| JP2455 | GCAAAATCAAGCGAACGG | ACT7 |
| JP9836 | GTGGCCGTGATCGGACTA | AT1G12160 |
| JP9837 | CAACGCTAACCGAGTCTGAA | AT1G12160 |
| JP9842 | GGTCGTGGCTTTGTTCAAGATA | AT1G31290 |
| JP9843 | GCCTTGACTCACTTGAGCTTG | AT1G31290 |
| JP9838 | CGGTGTTACAACTGGTGGAGT | AT3G22121 |
| JP9839 | CAAAACCTCCCATCGTAAAGC | AT3G22121 |
| JP9787 | TCGACTTGTTTGGACCTTGA | AT4G36510 |
| JP9788 | TCATGCGAATTATAGAAATTTAGACC | AT4G36510 |

Primers used for RT-qPCR:

| **PRIMER NAME** | **SEQUENCE** | **GENE** |
| --- | --- | --- |
| JP2452 | TCGTGGTGGTGAGTTTGTTAC | ACT7 |
| JP2453 | CAGCATCATCACAAGCATCC | ACT7 |
| JP9693 | AGAAATCTTCGACGCGGTCGTG | AT1G12160 |
| JP9694 | TCCCAGGAATATGAGCAAGACGAG | AT1G12160 |
| JP9721 | TCTCACACCGCTAGTGGTTCTC | AT1G31290 |
| JP9722 | TCAGGACGCTTTACTGGTTCTTTC | AT1G31290 |
| JP9709 | CGGTTGGTGGTTTAGGATGGGTAG | AT3G22121 |
| JP9710 | TCTCCTATGCTTGCGACTGTACC | AT3G22121 |
| JP9864 | GCTGTTTGAGTTCGCCGCCC | AT4G36510 |
| JP9865 | CCGACCAAAACTCCACCCGCC | AT4G36510 |
| JP9816 | TTCCGATTCACAGCGACCTAGC | AT3G12830 |
| JP9817 | TTGCTTCTTTGAGCGGCGAGTC | AT3G12830 |
| JP9949 | GCAAAGGGTTCGAGCTTCTTATGG | AT5G54490 |
| JP9950 | CGTCGATGCGTTTCTTCGTAAGC | AT5G54490 |
| JP9965 | GTTGTCACAAATTTCGCTGGCTTG | AT5G13320 |
| JP9966 | GCGCGTTGTTGTAGAAACCAGTC | AT5G13320 |
| JP2639 | CAGGCGAGCACACTGAACTG | Primer A Figure S12 |
| JP2640 | TCGTGGTCCGAAGAAAGGAA | Primer A Figure S12 |
| JP2695 | GGCGTATAGACCGATATGAGC | Primer B Figure S12 |
| JP2696 | TACTGCGTGGCACATTTGTT | Primer B Figure S12 |

Primers used for EMSA assays:

| **PRIMER NAME** | **SEQUENCE** | **PROBE** |
| --- | --- | --- |
| JP8487 | ACCAAGCAACACACCCCGT | UNSPECIFIC FWD |
| JP8493 | ACGGGGTGTGTTGCTTGGT | UNSPECIFIC REV |
| JP8489 | GTAGAATACTAGTTGATAAC | SPECIFIC FWD |
| JP8495 | GTTATCAACTAGTATTCTAC | SPECIFIC REV |

Primers used for BS-DNA amplification:

| **GENE** | **PRIMER NAME** | **PRIMER SEQUENCE** |
| --- | --- | --- |
| FWA | 2004 | GGTTTTATATTAATATTAAAGAGTTATGGGTYGAAGTTT |
|  | 2005 | CAAAATACTTTACACATAAACRAAAAACAAACAAATCRAA |
|  | 4423 | AACCAAAATCATTCTCTAAACAAAATATAAAAAAATC |
| Ta3 | 1269 | GAGAATYAGGTTAATAAGAAAGTGAAGTGTT |
|  | 1274 | CCACTRATTCCTRAAACACAACATTTCTRCTRATA |
| AtCOPIA4 | 3100 | GGTTGTYTGTGTTTTTTATGGTTYAGATTTTATA |
|  | 3101 | ATAACTRAACCACARATTCARACCCATTTTCATTT |
| AtSN1 | 1821 | CAATATACRATCCAAAAAACARTTATTAAAATAATATCTTAA |
|  | 1822 | GTTGTATAAGTTTAGTTTTAATTTTAYGGATYAGTATTAATTT |
